# Amine-Salt-Assisted Solution Crystallization of Inorganic Perovskite Single Crystals for High-Performance X-Ray Detection

**DOI:** 10.1007/s40820-026-02272-y

**Published:** 2026-07-01

**Authors:** Yujia Jiang, Depeng Chu, Jiacheng Pi, Naiming Liu, Xueni Sun, Yunxia Zhang, Ziyang Feng, Yuquan Wang, Wenjie Chen, Xinxin Wang, Jingyun Tian, Lei Zhao, Binxia Jia, Ruixin Shi, Yihui He, Yucheng Liu, Shengzhong Frank Liu

**Affiliations:** 1 Key Laboratory of Applied Surface and Colloid Chemistry Ministry of Education, Shaanxi Provincial Basic Discipline (Surface and Interface Chemistry) Research Center, Shaanxi Key Laboratory for Advanced Energy Devices, Shaanxi Engineering Lab for Advanced Energy Technology, School of Materials Science and Engineering, Shaanxi Normal University, Xi’an, 710119 People’s Republic of China; 2 School of Science, Xi’an University of Posts & Telecommunications, Xi’an, 710121 People’s Republic of China; 3 State Key Laboratory of Radiation Medicine and Protection, School of Radiation Medicine and Protection and Collaborative Innovation Center of Radiation Medicine of Jiangsu Higher Education Institutions, Soochow University, Suzhou, 215123 People’s Republic of China; 4 State Key Laboratory of Catalysis, Dalian National Laboratory for Clean Energy, Dalian Institute of Chemical Physics, Chinese Academy of Sciences, Dalian, 116023 People’s Republic of China; 5 Center of Materials Science and Optoelectronics Engineering, University of Chinese Academy of Sciences, Beijing, 100049 People’s Republic of China

**Keywords:** Perovskite, CsPbCl_3_ single crystal, Solution growth, X-ray detection

## Abstract

Inorganic perovskite CsPbCl_3_ single crystals (SCs) are promising for stable and high-performance X-ray detection due to their high X-ray absorption, superior optoelectronic properties, and superior chemical and thermal stability. However, the size of CsPbCl_3_ SCs grown via low-energy-consumption solution methods remains limited to below 1 mm, primarily because of the limited solubility of raw materials in solvents and the lack of effective growth techniques. In this work, an amine-salt-assisted solution crystallization strategy is developed to grow high-quality CsPbCl_3_ SCs with size up to 15 mm. This significant size improvement is enabled by a 13-fold increase in precursor solubility, achieved by constructing hydrogen bond interactions between the amine salt and insoluble raw materials. Consequently, the SCs exhibit high *μτ* product (6.3 × 10^–3^ cm^2^ V^−1^), low trap density (2.9 × 10^10^ cm^−3^), and large resistivity (1.7 × 10^9^ Ω cm). Therefore, the SC detectors achieve a record-high sensitivity of 76,624 μC Gy^−1^ cm^−2^ and a low detection limit of 47.9 nGy s^−1^ even under low bias voltages, enabling high contrast X-ray imaging. This ingenious and executable low-temperature solution crystallization strategy combined with superior X-ray detection performance makes CsPbCl_3_ SCs promising to advance the low-cost development of safe radiation detection systems.

**Supplementary Information:**

The online version contains supplementary material available at 10.1007/s40820-026-02272-y.

## Highlights

- An amine-salt-assisted solution crystallization strategy is developed to grow high-quality CsPbCl_3_ single crystals (SCs) with a size as large as 15 mm, the SCs exhibit a high carrier mobility-lifetime product, low trap density, and large resistivity.
- The CsPbCl_3_ SC X-ray detector achieved a high detection sensitivity of 76,624 μC Gy^-1^ cm^-2^ and a low detection limit of 47.9 nGy s^-1^, a short response time of 700 μs, therefore realized high contrast X-ray imaging.

**Supplementary Information:**

The online version contains supplementary material available at 10.1007/s40820-026-02272-y.

## Graphical abstract

**Figure:**
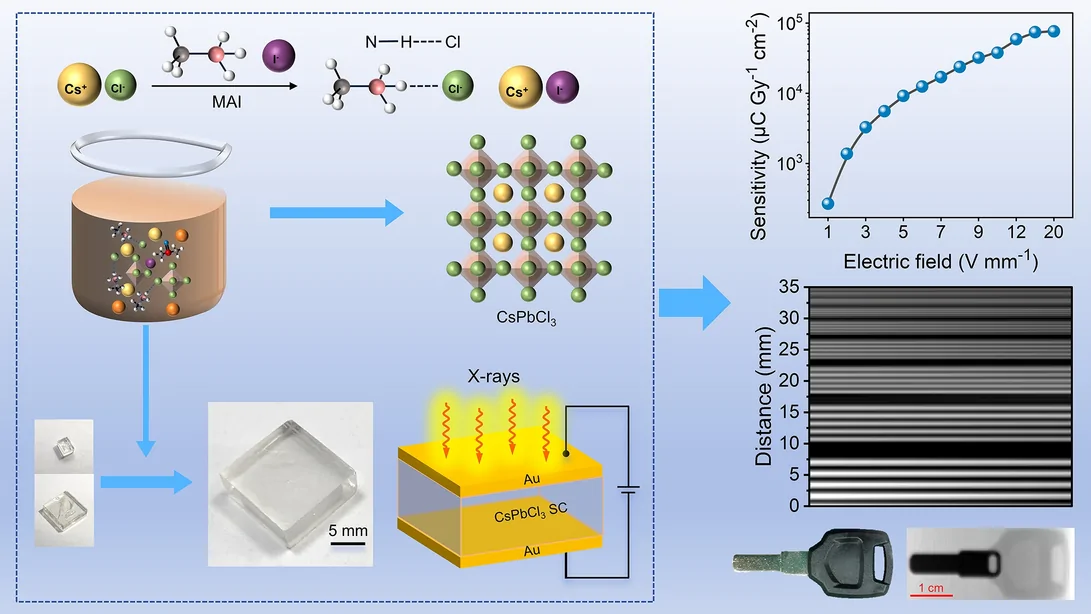

## Introduction

Metal halide perovskites have emerged as promising candidates for photovoltaic and photoelectronic applications due to their exceptional optoelectronic properties. Perovskite solar cells and detectors have made great strides in efficiency thanks to the inherent advantages of perovskites, including high charge carrier mobility, long carrier lifetime, adjustable crystal structure and composition, low defect density, and compatibility with low-cost solution processing [1–10]. These advantages also make them a prioritized choice for researchers in photovoltaic and optoelectronic applications. As a promising candidate, all-inorganic perovskites better suited to detect X-rays with high energy and strong penetration because the atomic numbers of Cs and Pb are 55 and 82, respectively. Among the CsPbX_3_ (X = Cl, Br, I) group, CsPbI_3_ is unsuitable for X-ray detection due to its destructive phase transition to yellow perovskitoid phase [11]. In contrast, the bromide perovskite CsPbBr_3_ SCs with high crystalline purity and large size have been successfully grown, and the corresponding detectors achieved both high resistivity and high mobility-lifetime (*μτ*) products, and demonstrated excellent detection performance under hard X-rays [12, 13] and *γ*-rays [14, 15]. In a previous report, CsPbCl_3_ crystals have been implemented in γ-ray detector by using high energy-consuming Bridgman melt growth technique at high temperature exceeding 600 °C [16]. However, large-size inorganic chloride perovskite CsPbCl_3_ SCs have not yet been achieved using low-temperature solution method and have not been investigated as X-ray detection materials due to the lack of effective growth methods. CsPbCl_3_-based devices exhibit three main advantages for X-ray detection. Firstly, its heavy elements effectively absorb X-ray photons. Secondly, the low intrinsic carrier concentration and high bulk resistivity resulting from the wide bandgap effectively suppress dark current and noise levels in the device. Finally, its all-inorganic composition gives it superior thermal stability compared to organic–inorganic halide perovskites, which helps extend the thermal degradation lifetime of device.

As a prototypical perovskite material, CsPbCl_3_ has historically been investigated in the forms of quantum dots, polycrystalline thin films and microcrystals obtained by using solution-based processing [17–19]. However, the exploration of large-size CsPbCl_3_ SC growth by low-temperature (< 120 °C) solution method remained limited until recent years owing to the formidable challenges associated with the low solubility of cesium chloride salt. In 2019, Gui et al. demonstrated a spatially confined solution-phase synthesis of micron-sized (about 60 μm) CsPbCl_3_ SC [20]. Pan et al. further advanced by introducing a thermodynamic crystal reconstruction protocol to convert the microcrystalline film into a larger CsPbCl_3_ SC film with size smaller than 1 mm. Despite these methodological innovations, the inherently low solubility of CsPbCl_3_ in conventional solvents and the prohibitive costs of Bridgman growth have prevented large-scale photoelectronic applications of large-size SCs. In addition, the existing synthesis strategies produce SCs (such as platelets or thin films) that are still insufficient to meet the requirements of large-size SCs for X-ray detector fabrication. These limitations collectively prevent the comprehensive understanding of the intrinsic properties of CsPbCl_3_ SC and its exploratory research in emerging optoelectronic applications. Therefore, it is extremely urgent to further develop effective strategies to grow large-size CsPbCl_3_ SC by low-temperature solution for photoelectric devices, especially X-ray detectors.

In this work, we achieved the growth of high-quality centimeter-sized CsPbCl_3_ SCs in low-temperature solution for the first time by significantly increasing the solubility of raw materials by 13-fold for SC growth through solution composition engineering combined with organic amine-salt-assisted strategy. The organic amine salt in solution can form hydrogen bonds with the insoluble chloride inorganic salts and weaken their lattice energy, thus greatly increasing the solubility. Based on the above strategies, the grown CsPbCl_3_ SCs not only show excellent thermal stability, but also have superior photoelectric properties, such as low trap density, high resistivity, and large *μτ* product. The above high figure-of-merits enable us realized high detection sensitivity, fast response speed, and low detection limit based on the CsPbCl_3_ SC detector, thus achieving high contrast X-ray imaging. These results further demonstrate the great potential of all-inorganic semiconductor perovskite CsPbCl_3_ SCs with wide bandgap obtained by a low-temperature solution method for low-cost and high-yield X-ray detection systems.

## Experimental Section

### Chemicals and Regents

Dimethyl sulfoxide (DMSO, 99.9%) was purchased from Aladdin Reagent Ltd. Caesium chloride (CsCl, 99.9%) and lead chloride (PbCl_2_, 99.99%) were purchased from Macklin Reagent. Methylammonium iodide (MAI, 99.99%), methylammonium bromide (MABr, ≥ 99.99%), formamidinium bromide (FABr, 99.9%), and formamidinium iodide (FAI, ≥ 99.99%) were purchased from Xi’an Nengcai Photoelectronic Technology Co., Ltd. All chemicals and reagents were used as received without further purification.

### Growth of the CsPbCl_3_ SCs

The precursor solutions for SC growth were prepared by dissolving stoichiometric amounts of CsCl, PbCl_2_, and the respective organic amine salts (MAI, MABr, FABr, or FAI) in DMSO, maintaining a molar proportion of 2:3:2. Specially, for the growth of CsPbCl_3_ single crystals without additives, CsCl and PbCl_2_ were dissolved in 500 mL of DMSO with a molar ratio of 1:1, resulting in a solution with a molar concentration of 0.005 M. For the growth of single crystal with MAI, MABr, FABr, and FAI, the molar concentration of CsPbCl_3_ was controlled at 0.05 M and dissolved in 120 mL of DMSO. All mixtures were subjected to continuous stirring on a hotplate maintained at 50 °C until complete dissolution was achieved, yielding optically clear and homogeneous solutions. Each precursor solution was subsequently filtered through a 2-μm PTFE membrane filter prior to crystallization. All the SCs were grown using the inverse temperature crystallization method. The crystallization vessels were placed in a static air-heating oven preheated to 50 °C. The system was maintained under stable conditions to promote the growth of high-quality perovskite single crystals. The oven temperature was then subsequently increased from 50 to 120 °C at a controlled rate of 1 °C per day until the completion of crystal growth.

### Device Fabrication

The as-grown CsPbCl_3_ SCs were mechanically polished to a thickness of 1 mm to meet the requirements for detector fabrication. Semi-transparent gold (Au) electrodes with a thickness of 100 nm were subsequently deposited on both the top and bottom surfaces of the crystal by thermal evaporation. During electrode deposition, the SCs were fixed on the mask with the active detection area of 1 × 1 mm^2^. Following this process, the electrode-coated SCs were mounted onto a custom-designed circuit board, and the two opposite electrodes of the detectors were stably connected to the corresponding terminals on the board.

## Results and Discussion

### Crystallization Processes Analysis of the SCs

**Fig. 1:**
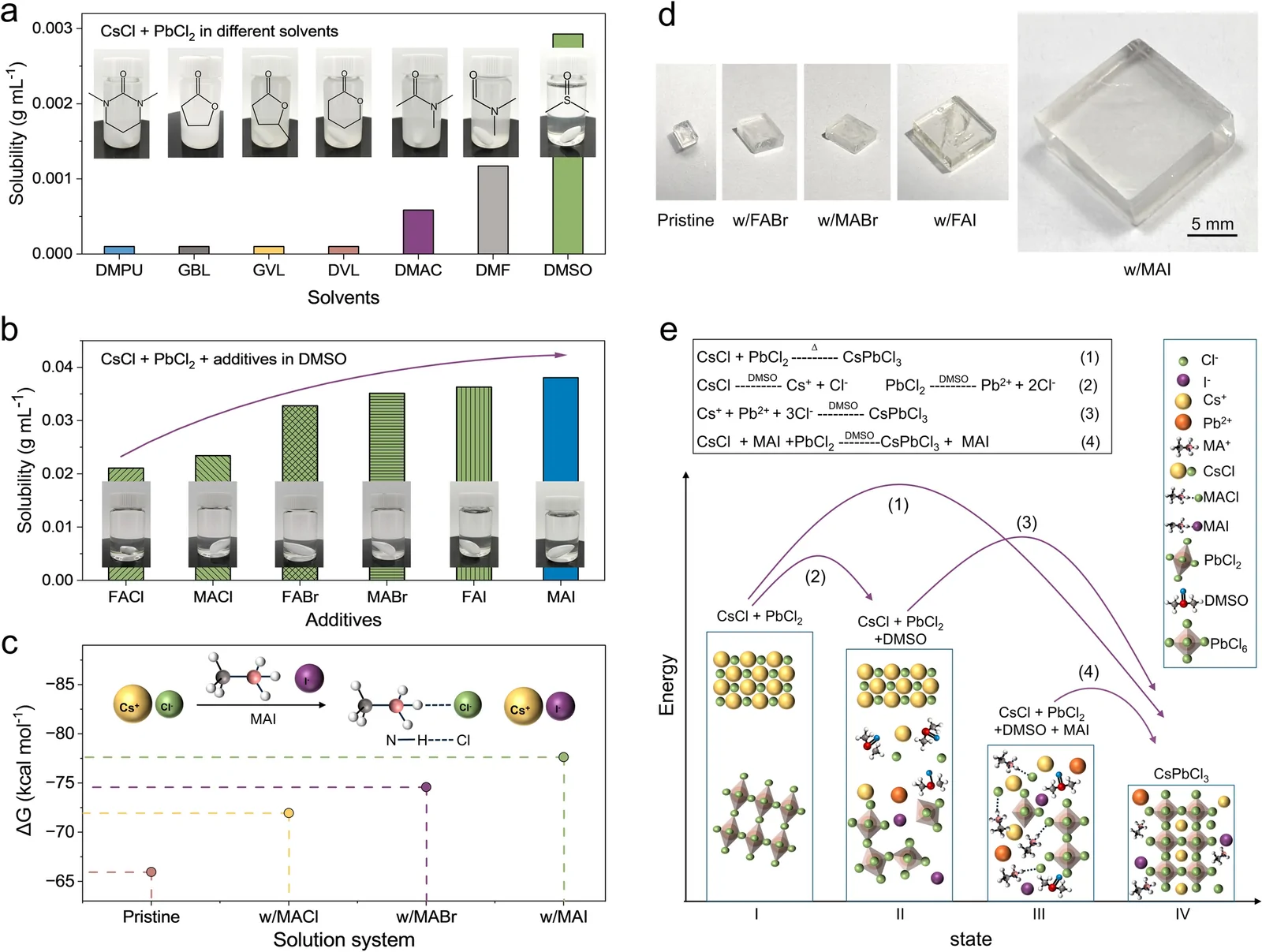
Growth mechanism of CsPbCl_3_ SC. **a** Solubility of the raw material CsCl and PbCl_2_ in various solvents at 25 °C. The inserted photographs show the dissolution situation of the raw materials dissolved in the solvents, with an amount of 2.93 × 10^–3^ g mL^−1^. **b** Solubility of the raw material CsCl and PbCl_2_ in DMSO at 25 °C with different additives added. The inserted photographs show the dissolution situation of the raw material at its corresponding solubility after using different additives. **c** Calculated binding Gibbs free energy change (ΔG) of the solution systems with different additives. Insert: illustration of the increase for the solubility when MAI is added to the solution. **d** Photographs of the CsPbCl_3_ SCs grown from the pristine solution and the solution with additives. **e** Growth mechanism of CsPbCl_3_ SCs in the pristine solution and the solution with MAI

As previously reported, limited by the low solubility of SC growth raw materials (CsCl and PbCl_2_) in solvents, there has been no substantial progress in the growth of large-size CsPbCl_3_ SCs in recent years. To address this limitation, we systematically explored the dissolution characteristics of different organic solvents for the SC growing raw materials at room temperature, the solvents including 1,3-dimethyl-3,4,5,6-tetrahydro-2 (1H)-pyrimidinone (DMPU), *γ*-butyrolactone (GBL), *γ*-valerolactone (GVL), *δ*-valerolactone (DVL), N, N-dimethylacetamide (DMAC), N, N-dimethylformamide (DMF), and dimethyl sulfoxide (DMSO). As shown in Fig. 1a, even in the popular highly soluble solvent DMSO, the solubility of the raw materials is only 2.93 × 10^–3^ g mL^−1^. In addition, the inserted photographs also indicates that when the same amount of raw materials (2.93 × 10^–3^ g mL^−1^) was added to a certain volume of solvent, it can be completely dissolved only in the solvent DMSO. However, this low solubility is still not sufficient for growing large-size SCs because the limited raw materials in solution does not continuously provide the necessary components for crystal growth. In fact, according to our experimental measurements, the low solubility of the raw materials used to grow CsPbCl_3_ SCs in DMSO is mainly caused by the insolubility of CsCl, while the solubility of PbCl_2_ is high enough (Fig. S1). Therefore, the key to increasing the concentration of the solution used to grow CsPbCl_3_ SCs is to enhance the solubility of CsCl in solvent.

Therefore, in this work, in order to increase the concentration of SC growth solution, we innovatively introduced organic amine salt R-NH_3_X (X = Cl, Br, I) into the solution to weaken the lattice energy of CsCl by forming hydrogen bonds (N–H···Cl) between R-NH_3_^+^ ions and CsCl, thus improving its solubility. The available amine salts tested include formamidine hydrochloride (CH(NH_2_)_2_Cl, FACl), methylamine hydrochloride (CH_3_NH_3_Cl, MACl), formamidinium bromide (CH(NH_2_)_2_Br, FABr), methylammonium bromide (CH_3_NH_3_Br, MABr), formamidinium iodide (CH(NH_2_)_2_I, FAI), and methylammonium iodide (CH_3_NH_3_I, MAI), etc. As shown in Fig. 1b, the common organic amine salts R-NH_3_X can significantly increase the concentration of the solution. We note that because the I^−^ ions and Cs^+^ ions can form more soluble Cs^+^–I^−^ ion pairs, the introduction of R-NH_3_I yields a higher concentration solution than the introduction of R-NH_3_Br and R-NH_3_Cl. In addition, since the better solubility of PbCl_2_ in DMSO, we appropriately increased the proportion of PbCl_2_ in the solution (PbCl_2_:CsCl = 3:2), which will be conducive to the raw materials can be crystallized according to the stoichiometric ratio of perovskite CsPbCl_3_ during the growth process. Combined with the introduction of MAI, we obtained the perovskite CsPbCl_3_ SC growth solution with an unprecedented concentration of (0.038 g mL^−1^), which is about 13-fold higher than the pristine concentration of 2.93 × 10^–3^ g mL^−1^. This is mainly because the introduced organic amine salt R-NH_3_X forms hydrogen bonds (N–H···Cl) with CsCl, which weakens the lattice energy of CsCl and thereby increases its solubility. During this process, the organic amine salt R-NH_3_I undergoes anion exchange (Cl⁻ substitution), resulting in the formation of Cs^+^I^−^:

$$CsCl^{ + } R - NH_{3}^{ + } I^{ - } \to Cs^{ + } I^{ - } + R - NH_{3}^{ + } Cl^{ - }$$

Furthermore, due to the combined effect of the reduced lattice energy of iodide salts (CsI, which is more soluble in DMSO than CsCl) and the solvation effect, the highest solution concentration was achieved after adding MAI to the SC growth solution.

We further studied the dissolution process of the growth solution using molecular dynamics simulations, and calculated the total electronic energy at the optimized geometry and Gibbs free energy (*G*) of the solvent molecule, the raw materials (Table S1), and the SC growth solutions (Table S2) with different compositions constructed by the solvent molecule and the raw materials. In the solution system, the oxygen atoms of DMSO molecules coordinate with Pb^2+^ in solution through their polar ligands to form a relatively stable coordination structure, while Cs^+^ ions mainly coordinate with halogen anions and few DMSO molecules (Fig. S2). When the R-NH_3_I is introduced in the solution, the –NH_3_^+^ group will form hydrogen bonds with CsCl, thus changing the coordination structure and improving its solubility. In addition, the electrostatic interactions between Pb-halide and Cs-halide would be reduced due to the weak electronegativity of I^−^, thus increasing the total energy of the solution system. By subtracting the respective Gibbs free energy of each solvent molecule and the raw material from the total energy of the solution system, we obtain the binding Gibbs free energy change (ΔG) of the solution system, which can also be considered as the binding energy between the solute and the solvent in the solution (Table S3). Figure 1c shows the comparison of the binding energy between solute and solvent molecules in different solution systems, where (CsCl-PbCl_2_-DMSO-MAI) < (CsCl-PbCl_2_-DMSO-MABr) < (CsCl-PbCl_2_-DMSO-MACl) < (CsCl-PbCl_2_-DMSO), which is consistent with the experimental results of solubility. In general, the more negative the change of Gibbs free energy in the dissolution process (ΔG < 0), the easier the binding (stronger interaction) between solute and solvent molecules is, and the more conducive it is to form a solution system with high solubility.

Under the guidance of the above design, we grew CsPbCl_3_ SCs by introducing MAI into the SC growth solution and combining the inverse temperature crystallization (ITC) method developed by our group, the detailed growth method is described in the experimental section. As shown in Fig. 1d, after the same growth time, we only obtained CsPbCl_3_ SCs with regular shape in pristine solution and the solutions with FABr, MABr, FAI, and MAI, but no satisfactory crystals were obtained in the solutions with MACl and FACl (Fig. S3). We note that the higher the solution concentration, the larger the crystal size, in which the SC grown in the solution with MAI reached 15 × 15 × 6 mm^3^, and the SC shows smooth surface, high transparency and no cracks. In addition, the SCs grown in the pristine solution are not only small in size, but also contain obvious cracks inside (Fig. S4). To the best of our knowledge, this is the first case in which centimeter-sized CsPbCl_3_ SCs have been successfully grown by solution method at low temperature (< 120 °C).

The growth mechanism of CsPbCl_3_ SCs in the pristine solution and the solution with additives is illustrated in Fig. 1e. Compared to the direct growth of CsPbCl_3_ SCs by Bridgman method at high temperature (Eq. 1 in Fig. 1e), the energy barrier that needs to be overcome to grow in solution is significantly reduced. The energy barrier of solid-state reaction crystallization between CsCl and PbCl_2_ (state I) is generally higher than that of solution reaction crystallization, which is mainly limited by the difficulty of solid-state diffusion and the high consumption of lattice energy. Because the solution environment significantly reduces the activation energy required for the reaction through the solvent medium and the kinetic advantage. This difference also explains why solid-state reaction crystallization (Bridgman method) requires high temperature conditions, while solution reaction crystallization can be carried out under mild conditions.

For the solution method, the formation of supersaturated solution is a prerequisite for driving crystallization, and obtaining a sufficiently high solution concentration is an important basis for realizing the growth of large-size SCs. Therefore, it is necessary to ensure that the raw materials are fully dissolved in the solvent. Our previous study showed that only a small fraction of CsCl dissociates into free Cs^+^ and Cl^−^ ions or forms complexes with DMSO to dissolve (state II) due to the very limited solubility of CsCl in DMSO solvent (Eq. 2 in Fig. 1e). Due to the insufficient availability of solute ions, growing CsPbCl_3_ SCs in the DMSO solution is extremely challenging (Eq. 3 in Fig. 1e). We attempted to grow CsPbCl_3_ SCs in the pristine solution with extremely low concentrations, but the results showed that these SCs contain obvious defects from the surface to the interior (Fig. S4), which hindered them from exhibiting superior photoelectric performance characteristics that high-quality SCs should possess.

Upon introducing MAI to the SC growth solution, the hydrogen bond interaction between MA^+^ ions and CsCl in the solution significantly enhanced the solubility of the SC growth raw materials in DMSO (State III), thereby obtaining a high-concentration SC growth solution with sufficient ions of Cs^+^, Pb^2+^, and Cl^−^. As the temperature increases, the solution gradually becomes supersaturated, thereby driving the crystallization of CsPbCl_3_ (State IV). The calculated energy values corresponding to states I, II, and III are given in Table S2, and the energy relationship among these three states is *E*_(State I)_ > *E*_(State II)_ > *E*_(State III)_. It is worth noting that the externally introduced MA^+^ and I^−^ ions did not enter the final CsPbCl_3_ SC lattice. This is confirmed in subsequent studies and is consistent with the results reported by Ji et al. [21].

### Structure and Properties Analysis of the SCs

**Fig. 2:**
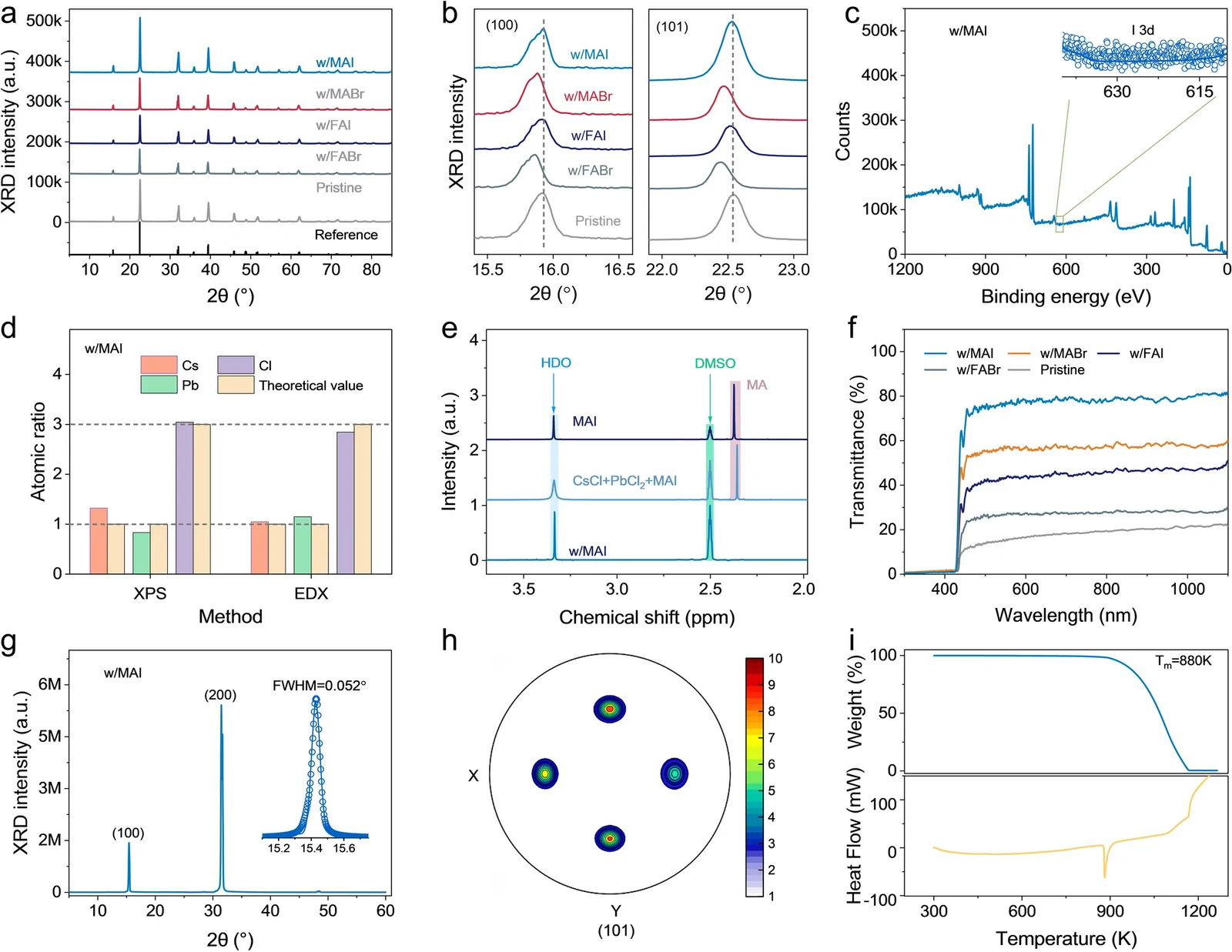
Characterization of the CsPbCl_3_ SCs. **a** Powder XRD patterns of the SCs grown from the pristine solution and the solution with different additives. **b** Enlarged views of the diffraction peaks of the (100) and (101) plane in Fig. 2a. **c** XPS spectrum of the CsPbCl_3_ SC grown from the solution with MAI. **d** Comparison of calculated Cs:Pb:Cl ratio in the CsPbCl_3_ SC with experimental results measured by XPS and EDS, respectively. **e** ^1^H-NMR spectra of the CsPbCl_3_ SC powder, a mixture of CsCl, PbCl_2_ and MAI, and pure MAI dissolved in DMSO-d_6_. **f** Transmittance spectra of the SCs. **g** High-resolution XRD rocking curve of the CsPbCl_3_ SC. **h** Pole figure of the CsPbCl_3_ SC measured at the (101) plane. **i** Thermogravimetric and differential scanning calorimetry analysis of the CsPbCl_3_ SC

X-ray diffraction (XRD) is an effective method for analyzing the crystal structure and crystalline quality of semiconductor materials. As shown in Fig. 2a, the XRD patterns of SCs grown from the pristine solution and the solution with added MAI, MABr, FAI, and FABr were measured, and all of them exhibit similar diffraction peaks. Notably, the SC grown from the solution with MAI (w/MAI) demonstrated the highest diffraction peak intensity, indicating the best crystallinity. Furthermore, when the XRD patterns were magnified (Fig. 2b), it is observed that the XRD diffraction peaks of the SCs grown in the solutions with MAI and FAI remained consistent with those of the pristine SC, indicating that the lattice had not changed. This proved that the I^−^ ions and MA^+^ ions did not enter the CsPbCl_3_ SC lattice. However, the XRD diffraction peak positions of the SCs grown in the solutions with MABr and FABr shifted significantly toward lower angles, suggesting that the Br^−^ ions have been incorporated into the SC lattice, thereby causing lattice expansion.

To further verify the specific components of the SCs grown in the solution containing additives, X-ray photoelectron spectroscopy (XPS) measurements were performed on the internal cross section of the fresh SCs. As shown in Fig. 2c, no XPS signal from I^−^ ion was detected in the CsPbCl_3_ SC grown from the solution with MAI, which is consistent with the result that the XRD diffraction peak position remained unchanged. Furthermore, we conducted XPS measurements on the SCs grown in multiple batches, which further confirmed this result. Subsequently, we performed XPS measurements on the SCs grown in multiple batches, thereby verifying the reliability of this result (Fig. S5). The specific composition of the CsPbCl_3_ SC was analyzed by both XPS and energy-dispersive X-ray spectroscopy (EDX) attached to the scanning electron microscopy (Fig. S6). No cracks or grain boundaries are observed in the SEM image, which proved the structural integrity and continuity of the SC. EDX mapping indicated that the Cs, Pb, and Cl in the SC is uniformly distributed, which further confirmed the high quality of the SC. As shown in Fig. 2d, the Cs:Pb:Cl ratio determined by the experiments was close to the stoichiometric ratio of 1:1:3 in the perovskite CsPbCl_3_. We also conducted detailed XPS and EDX analyses on the SCs grown from solutions with different additives. The results showed that no I^−^ ions were detected in the SCs grown from the solution containing FAI (Fig. S7), while Br^−^ ions could be clearly identified in the SCs grown from the solutions with MABr (Fig. S8) and FABr (Fig. S9). These results further demonstrated that the I^−^ ions in the solution did not enter the CsPbCl_3_ SC, but the Br^−^ ions did. The Br^−^ ions can enter the CsPbCl_3_ lattice mainly due to its moderate ionic radius (~ 196 pm), similar bonding characteristics to Cl^−^ (~ 181 pm), and the thermodynamically stable solubility formation ability. However, I^−^ ions (~ 220 pm) are difficult to stably replace the Cl^−^ lattice position in the CsPbCl_3_ due to their large ionic radius, weak bonding, and tendency to cause lattice distortion, These results are in agreement with the conclusions of the XRD analysis, and this similar phenomenon has also been observed in previous reported works [22–24]. Furthermore, the 1H NMR spectroscopy measurements indicate that there is no MA^+^ ion present in the CsPbCl_3_ SC (Fig. 2e), confirming that MAI had not been incorporated into the SC lattice.

For wide-bandgap semiconductor crystals, the optical transmittance is a direct indicator for measuring crystal quality and defect density, because a high defect concentration will enhance photon scattering and the capture of electromagnetic waves, thereby reducing the transmittance. Generally, crystals with high defect concentration usually have lower transmittance. The main reason is that defects (such as vacancies, dislocations, impurities, etc.) will disrupt the periodicity of the lattice, causing the incident light to be scattered or absorbed. Moreover, the stress field around the defects will cause non-uniform refractive index, further intensifying the scattering or distortion of the light. Therefore, a high defect concentration significantly reduces the optical transmittance of the SC, especially in the ultraviolet, visible, and near-infrared wavelength ranges, where the effect is more pronounced. As shown in Fig. 2f, among the SCs grown in solutions with different additives, the CsPbCl_3_ SC grown from the solution with MAI shows the highest optical transmittance, indicating its best crystal quality. To evaluate the crystallization quality of CsPbCl_3_ SC, we analyzed the SC using high-resolution XRD rocking curve. As shown in Fig. 2g, the FWHM of the rocking curve for the SC was measured to be 0.052°, further confirming its superior crystallization quality. Furthermore, by azimuthally rotating the SC from 0° to 360° at tilt angles ranging from 0° to 90° at a fixed 2θ, the pole figure was determined. As shown in Fig. 2h, the discrete diffraction points are arranged in a symmetrical square pattern, further confirming the high symmetry of the crystal structure [25]. Furthermore, the photoluminescence (PL) spectrum of the CsPbCl_3_ SC at 421 nm with an extremely small FWHM of 6.72 nm (Fig. S10) also indicates the high quality. These results collectively demonstrated the high quality of the CsPbCl_3_ SC. Subsequently, the SC was ground into powder for UV absorption spectrum analysis. As shown in Fig. S11, the UV absorption edge of the CsPbCl_3_ SC powder was measured to be 423 nm, which is consistent with the PL result. The thermal stability of this inorganic perovskite SC was further evaluated through thermogravimetric analysis and differential thermal analysis. As shown in Fig. 2i, the CsPbCl_3_ SC did not decompose until at a high temperature of 880 K, which is much superior to other organic–inorganic halide perovskites such as MAPbX_3_ and FAPbX_3_ [26, 27].

### Carrier Transport Properties Analysis of the SCs

**Fig. 3:**
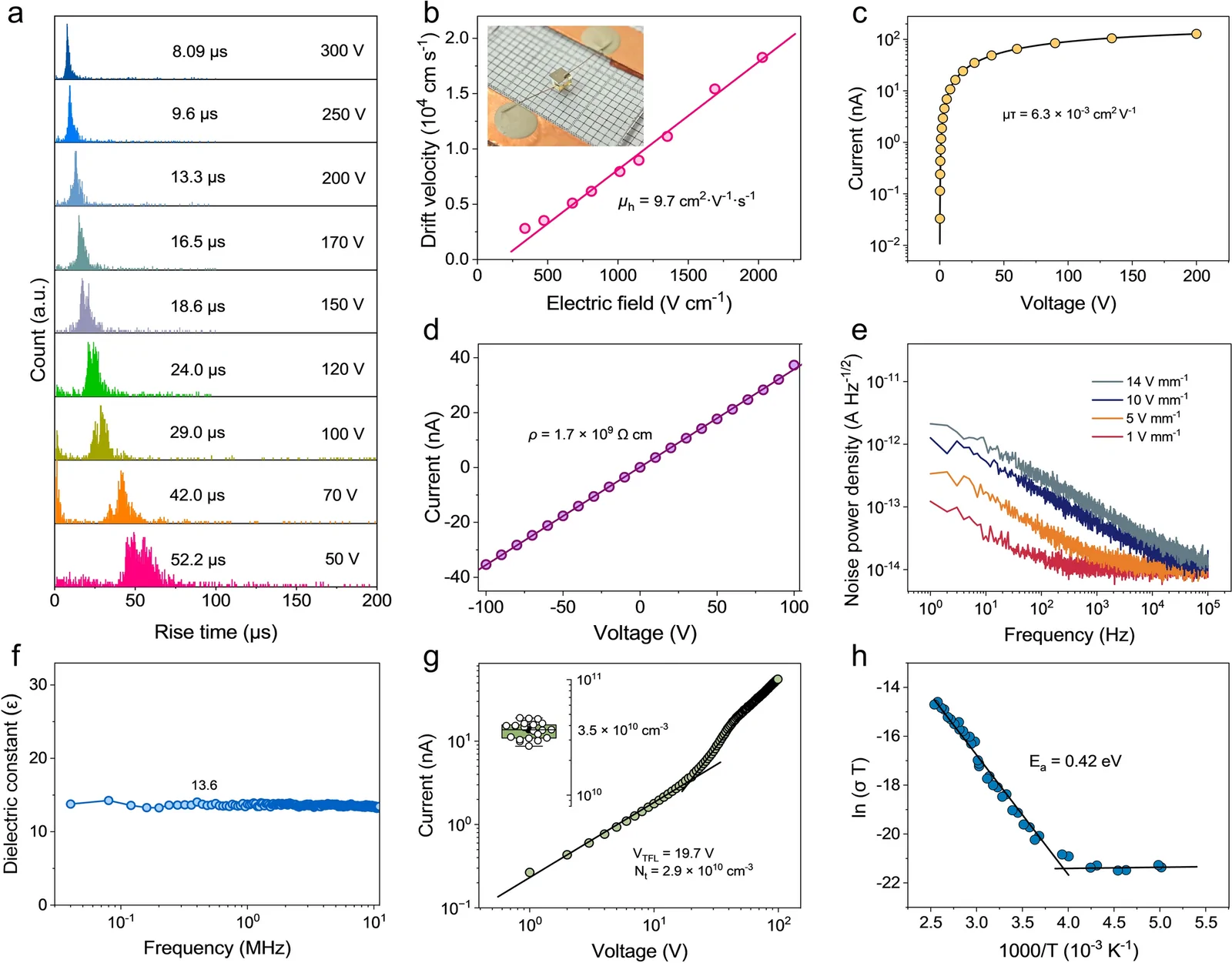
Electrical properties of the CsPbCl_3_ SCs. **a** Distribution of output preamplifier pulse rise times observed during the ^241^Am 5.5 MeV *α* particles measurement under various bias voltages. **b** Mobility of hole carriers estimated based on the pulse rise time. Insert: photograph of the SC device used for particles measurement. **c** Photoconductivity measurements of the CsPbCl_3_ SCs to determine the *μτ* products. **d** Resistivity measurements of the CsPbCl_3_ SCs. **e** Dark noise current of the CsPbCl_3_ SC device at different frequencies. **f** Dielectric constant measurements of the CsPbCl_3_ SC. **g** Trap density measurements of the CsPbCl_3_ SCs. Inset: statistics of the trap density for the CsPbCl_3_ SCs. **h** Temperature-dependent conductivity measurements of the CsPbCl_3_ SCs

The carrier mobility (*μ*) of the material is one of the key parameters affecting the performance of X-ray detectors, as it directly determines the transport and collection efficiency of carriers within the material. Figure 3a shows the rise time distribution of preamplifier pulses generated by ^241^Am α particles across varying applied voltages of the CsPbCl_3_ SC device. As the voltage increased from -50 to -300 V, the pulse rise time decreased significantly from 52.2 to 8.09 μs. The revealed rise time distributions become narrower with smaller average rise time at higher applied voltage. Therefore, hole mobility of the CsPbCl_3_ SC was extracted from the voltage-dependent rise time of pulse signals using the drift velocity relation *μ* = *d* / (*t* × *E*) [28, 29], yielding a value of 9.7 cm^2^ V^−1^ s^−1^, as shown in Fig. 3b. A high mobility-lifetime (*μτ*) product enables carriers to move a longer distance under the electric field, thereby improving the charge collection efficiency, enhancing the sensitivity and signal-to-noise ratio (SNR) of the CsPbCl_3_ SC detectors. In this work, we determined the *μτ* product of the CsPbCl_3_ SCs through photoconductivity characteristic measurements [30]. It can be seen that under X-ray irradiation, the *μτ* product of the SCs fitted from the photoconductivity curves is determined to be 6.3 × 10^–3^ cm^2^ V^−1^ (Fig. 3c), which is comparable to the previously optimized inorganic perovskite CsPbBr_2.9_I_0.1_ SC with a *μτ* product of 2.65 × 10^–3^ cm^2^ V^−1^ [31]. The high *μτ* product of the CsPbCl_3_ SCs will enable its X-ray detectors to achieve sufficient charge collection under a lower electric field and reducing the working voltage of the detector, thereby lowering power consumption and the risk of breakdown.

We also measured the bulk resistivity of the CsPbCl_3_ SC. As shown in Fig. 3d, the resistivity of CsPbCl_3_ SC grown using this solution strategy is 1.7 × 10^9^ Ω cm, which enables the SC devices to show a lower noise current (Fig. 3e). The traps in the SC will cause carrier recombination and serve as the main channels for ion migration. Therefore, they will significantly reduce the response and stability of the SC device. In this work, the SCLC measurements were performed to quantify the trap states density of the CsPbCl_3_ SCs. Combined with the experimentally measured dielectric constant (Fig. 3f), the trap density of the SC was determined to be 2.9 × 10^10^ cm^−3^ (Fig. 3g) from the dark current–voltage (I-V) curve with a 19.7 V trap-filled limit voltage (*V*_TFL_). Additionally, we conducted a statistical analysis of the trap density for 20 SCs (Fig. S12), revealing that the average trap density of the SCs is 3.5 × 10^10^ cm^−3^, which is nearly 22 times lower than that of previously reported CsPbCl_3_ SCs (7.6 × 10^11^ cm^−3^) [32]. This result indicates that the additive-assisted crystallization growth strategy in this work can effectively improve the quality of inorganic halide perovskite CsPbCl_3_ SCs. The low trap density is expected to help reduce the loss caused by the trap-induced recombination of photogenerated carriers and can suppress the leakage current in photoelectronic devices.

Ion migration will alter the local electrical properties of the X-ray detection material, such as creating space charge regions or introducing additional defect states, thereby interfering with the transport and collection efficiency of carriers [33]. This effect is particularly pronounced under high electric fields or high-dose X-ray irradiation, potentially causing baseline drift and signal attenuation in X-ray detectors, which can affect long-term stability and measurement accuracy. Moreover, ion migration may also trigger the polarization effect in X-ray detectors, forming an internal reverse electric field, weakening the effective detection electric field and reducing the charge collection efficiency. To assess the extent of ion migration in the CsPbCl_3_ SC, we conducted temperature-dependent conductivity measurements under dark conditions. Typically, at low temperatures, electron conduction plays a dominant role as ion migration is inhibited. In this study, the ion mobility in the CsPbCl_3_ SC was characterized by the activation energy (*E*a) of the ionic conductivity, which could be calculated by the Nernst–Einstein equation based on the temperature-dependent conductivity measurements [34]:

$$\sigma \left( T \right) = \frac{{\sigma_{0} }}{T}\exp \left( { - \frac{{E_{a} }}{{k_{B} T}}} \right)$$

where *k*_B_ the Boltzmann constant, *σ*₀ is a pre-exponential factor, and *T* is the absolute temperature. By fitting the temperature-dependent conductivity curve in the high temperature region, we determined the activation energy during ion migration in CsPbCl_3_ SC to be 0.42 eV (Fig. 3h). A higher *E*a indicates the lower ion migration in the CsPbCl_3_ SC, which will facilitate its SC detector to achieve stable X-ray response output and superior long-term operational stability.

### X-Ray Detection Performance of the SC Detectors

**Fig. 4:**
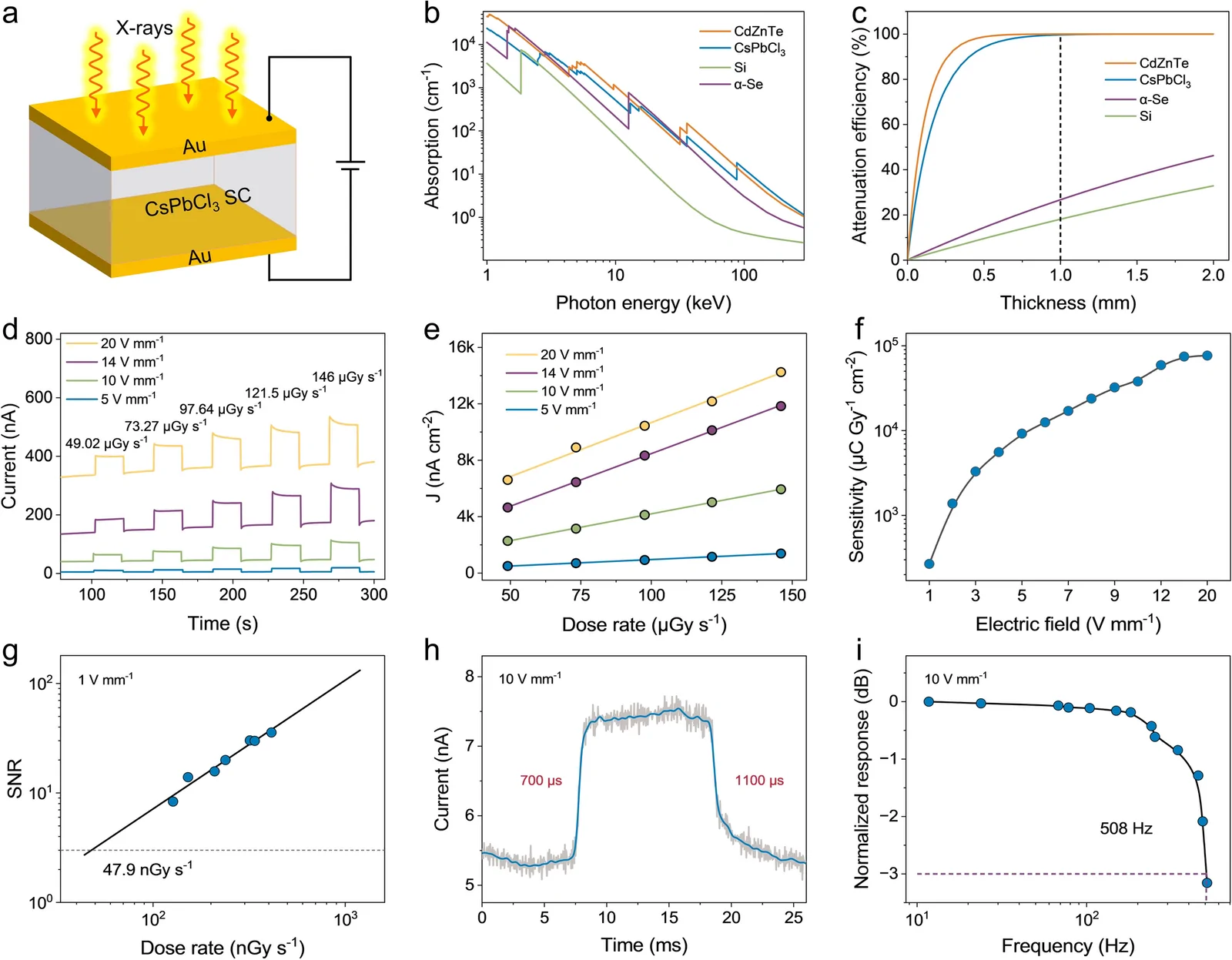
X-ray detection of the devices. **a** Structure of the CsPbCl_3_ SC detector. **b** X-ray absorption coefficients of CsPbCl_3_, CdZnTe, *α*-Se, and Si at different photon energies. **c** Thickness dependence attenuation of CsPbCl_3_, CdZnTe, *α*-Se, and Si to X-rays. **d** Response of the CsPbCl_3_ SC detector under X-rays. **e** Dose rate dependence response of the CsPbCl_3_ SC detector under X-ray irradiation at different electric fields. **f** X-ray detection sensitivity of the CsPbCl_3_ SC detector at different electric fields. **g** Response SNR of the CsPbCl_3_ SC detector under different X-ray dose rates. **h** Transient response of the CsPbCl_3_ SC detector to pulsed X-rays at a fixed electric field of 10 V mm^−1^. **i** Normalized response of the CsPbCl_3_ SC detector under X-rays with different modulation frequencies

Given its high *μτ* product, low trap, large resistivity, suppressed ion migration and strong X-ray absorption, the inorganic halide perovskite CsPbCl_3_ SCs are expected to exhibit superior X-ray response. Therefore, the CsPbCl_3_ SCs were fabricated into detectors with a structure of Au/CsPbCl_3_ SC/Au as illustrated in Fig. 4a. During the detector preparation, we firstly evaluated the X-ray absorption properties of the CsPbCl_3_ SC, and found that its X-ray absorbance coefficient is higher than that of traditional X-ray detection materials (such as Si and *α*-Se), as shown in Fig. 4b. Considering the correlation between the absorption coefficient, X-ray penetration depth and X-ray energy conversion efficiency [35], we further calculated the thickness-dependence X-ray attenuation of the CsPbCl_3_ SC to X-rays with specific energy. As shown in Fig. 4c, it can be seen that a 1-mm-thick CsPbCl_3_ SC can almost completely (99.8%) absorb the X-rays (70 kVp) used in the work. Therefore, we prepared the CsPbCl_3_ SC detectors with a thickness of 1.0 mm, and measured their X-ray detection performance.

As shown in Figs. 4d and S13a, the response signal of the CsPbCl_3_ SC detector gradually enhanced with the increase in X-ray dose rate under different electric fields, indicating sensitive response to X-rays. Moreover, the detector shows a stable dark current baseline, further confirming the weak ion migration of the CsPbCl_3_ SC. By fitting the response current density of the SC detector under different dose rates of X-ray irradiation (Figs. 4e and S13b), the X-ray detection sensitivity was determined. As shown in Fig. 4f, the SC detector achieved a sensitivity as high as 76,624 μC Gy^−1^ cm^−2^ only at a low electric field of 20 V mm^−1^, which is mainly attributed to the high *μτ* product of the CsPbCl_3_ SC. This value represents record-high sensitivity for inorganic perovskite SC detectors, underscoring their significant application potential (Table S4). Detection limit is usually regarded as the X-ray dose rate that the detector can effectively detect and generate a response with SNR = 3 [36]. Therefore, we measured the response of CsPbCl_3_ SC detector at low dose rates, and 47.9 nGy s^−1^ was determined as the detection limit at 1 V mm^−1^ (Fig. 4g).

Response time is a crucial parameter for X-ray detectors. Achieving a short X-ray response time, combined with high detection sensitivity, provides an effective pathway to significantly reduce the X-ray dose required for imaging. In this work, the response rise time (*t*_r_) of the X-ray detector is defined as the time required for the response value to increase from 10 to 90% of its peak value, while the fall time (*t*_f_) is defined as the time for the response to decrease from 90 to 10% of the peak value [37]. As shown in Fig. 4h, the rise time of the CsPbCl_3_ SC detector response to X-rays was measured to be 700 μs, and the fall time was 1100 μs. Additionally, we further analyzed the response speed of the SC detector by measuring the −3 dB cutoff frequency (*f*_3dB_) of the X-ray response. The response cutoff frequency of the detector is defined as the modulation frequency of the input signal when its amplitude remains constant and the output response signal drops to 0.707 times its maximum value. In this study, the −3 dB cutoff frequency of the SC detector was determined to be 508 Hz (Fig. 4i) according to the relationship between the response amplitude and the modulation frequency [38]:

$$f_{3dB} = \frac{0.35}{{t_{r} }}$$

where *t*_r_ is the response time, and it is determined to be 689 μs, which is consistent with the response time of 700 μs obtained from the transient response measurement.

### Stability and X-Ray Imaging Performance of the SC Detectors

**Fig. 5:**
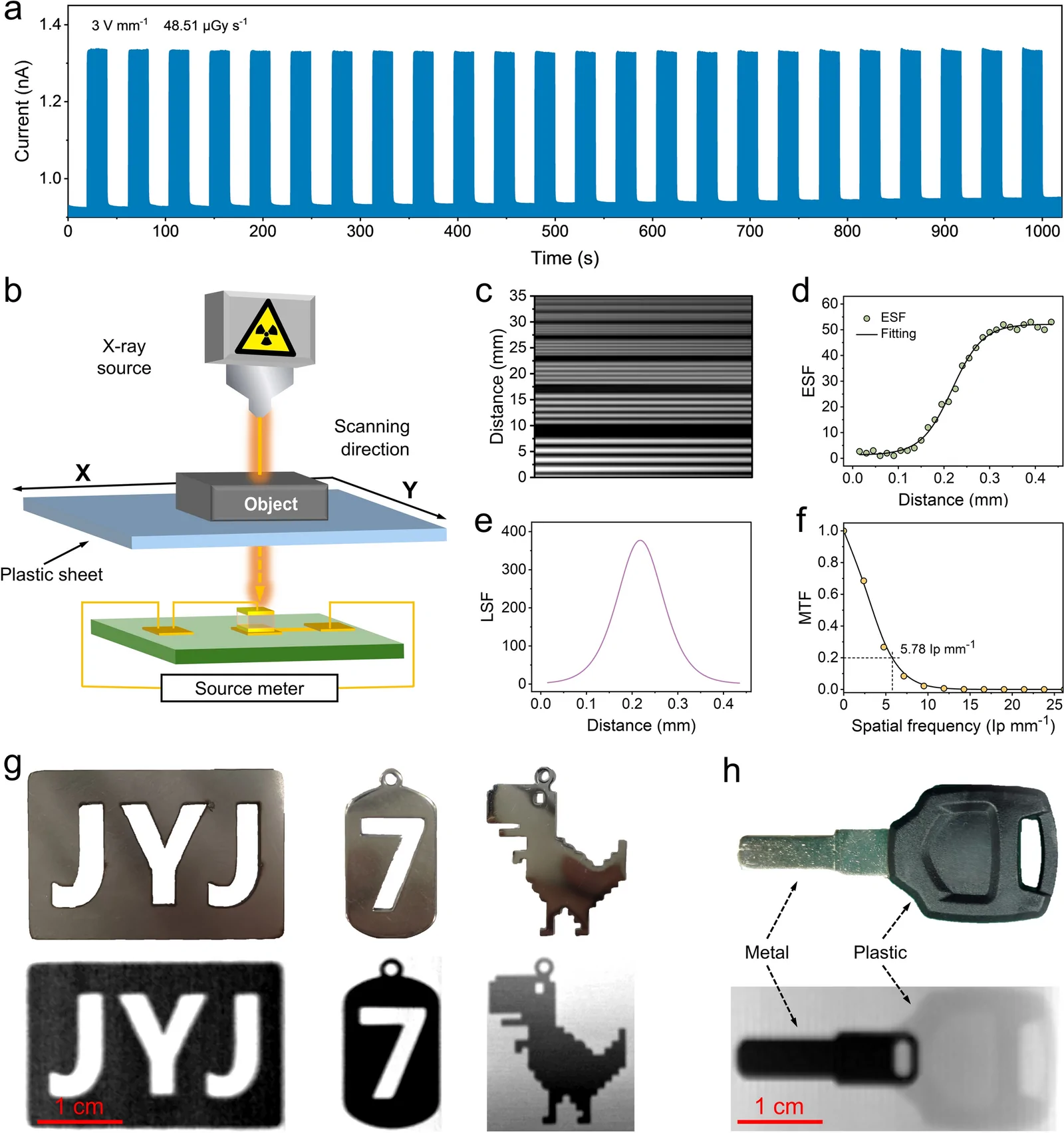
Stability and X-ray imaging of the CsPbCl_3_ SC detector. **a** Response currents of the CsPbCl_3_ SC detector under on–off X-rays with a dose rate of 47.9 μGy s^−1^ and at an electric field of 3 V mm^−1^. **b** Illustration of imaging method on the CsPbCl_3_ SC detector. **c** X-ray image of a standard line pair card measured by the SC detector. **d–f** Edge spread function (ESF), line spread function (LSF), and modulation transfer function (MTF) of the CsPbCl_3_ SC detector. **g** Photographs and the corresponding X-ray images of a “dinosaur” pattern, the number “7”, and the letter metal cards of “JYJ”. **h** Photograph and X-ray image of a key

The stability of halide perovskite photoelectric devices has long been a key research focus in this field. To assess the stability of the CsPbCl_3_ SC detector under X-ray irradiation, we carefully recorded the response signal of the detector during continuous operation under a specific dose rate of 48.51 μGy s^−1^ and an applied electric field of 3 V mm^−1^. As shown in Fig. 5a, both the dark current and photocurrent of the detector exhibit negligible changes throughout the whole evaluation period, which demonstrates the low ionic migration of the CsPbCl_3_ SC. Furthermore, we evaluated the environmental stability of the CsPbCl_3_ SC detector. As shown in Fig. S14, the detector still maintained 85% of its response to X-rays after being placed in the air for 35 days. The superior X-ray response sensitivity, low dark current noise and stable response output make the CsPbCl_3_ SC detector promising for achieving high-resolution X-ray imaging. In this work, we constructed an X-ray imaging measurement system as illustrated in Fig. 5b to verify the imaging performance of the SC detector. We firstly conducted an X-ray scan on a standard line pair card, and the X-ray image shows that even very narrow metal (Pb) lines could be clearly identified (Fig. 5c). Specifically, the line pair card was installed on the scanning stage between X-ray source and the CsPbCl_3_ SC detector. During the movement of the stage, the X-ray response signal of the detector and the coordinate position of the object were simultaneously collected, thereby forming a two-dimensional X-ray image. Furthermore, the modulation transfer function (MTF) was measured by an edge device scanning method [39], and the corresponding resolution of the CsPbCl_3_ SC detector was determined to be 5.78 lp mm^−1^ (Fig. 5d–f). Therefore, we performed X-ray imaging on a “dinosaur” pattern, the number “7”, and the letter metal cards of "JYJ", as shown in Fig. 5g. The X-ray images of these objects are consistent with the physical photographs. More importantly, the CsPbCl_3_ SC detector can clearly identify the metal inside the black plastic of the key (Fig. 5h). The high-resolution X-ray images further confirm that the CsPbCl_3_ SC detectors possess sensitive X-ray response and high spatial resolution.

## Conclusion

In this work, by constructing hydrogen bond interactions between organic amine salts and insoluble growth raw materials and combining solution component engineering, the solubility of CsPbCl_3_ was increased by 13-fold, thereby solving the solubility limitation of inorganic halide perovskite CsPbCl_3_ in organic solvents. Thus, an organic amine-salt-assisted solution crystallization strategy was successfully developed to achieve the first growth of large-sized and high-quality CsPbCl_3_ SCs. The obtained CsPbCl_3_ SCs exhibit superior photoelectric properties, such as low defect density, large resistivity, high carrier-lifetime product, and low ion migration. Therefore, X-ray detectors based on CsPbCl_3_ SCs realized record-high detection sensitivity among inorganic perovskite X-ray detectors even under low electric fields, as well as low detection limit, short response time, and stable response output. The rare combination of these exceptional X-ray detection properties enables the CsPbCl_3_ SC detectors achieved high-definition X-ray imaging. It can be expected that through further optimization of SC quality and integration of advanced high-pixel optoelectronic device technologies, the imaging performance of CsPbCl_3_ SC detectors will be further improved. In conclusion, this work lays an important foundation for developing a new generation of X-ray detection equipment with stable and high-resolution imaging capability and provides a new solution for achieving safe and high-frequency medical imaging applications in the future.

## Supplementary Information

Below is the link to the electronic supplementary material.

Supplementary file1 (DOCX 37262 KB)
