# Supplementary material for: Amine-Salt-Assisted Solution Crystallization of Inorganic Perovskite Single Crystals for High-Performance X-Ray Detection
Source: Nanomicro Lett. 2026 Jul 1;18:421. doi: 10.1007/s40820-026-02272-y (PMC13323420; doi:10.1007/s40820-026-02272-y)
Supplement: Supplementary file 1 — Supplementary file1 (DOCX 37262 KB) [file 40820_2026_2272_MOESM1_ESM.docx]

Supporting Information for

**Amine-Salt-Assisted** **Solution** **Crystallization of Inorganic Perovskite Single Crystals for High-Performance X-ray Detection**

Yujia Jiang^1#^, Depeng Chu^1#^, Jiacheng Pi^1^, Naiming Liu^1^, Xueni Sun^1^, Yunxia Zhang^2^, Ziyang Feng^1^, Yuquan Wang^3^, Wenjie Chen^3^, Xinxin Wang^3^, Jingyun Tian^1^, Lei Zhao^1^, Binxia Jia^1^, Ruixin Shi^1^, Yihui He^3^*, Yucheng Liu^1^*, Shengzhong (Frank) Liu^1,4,5,^*

^1^Key Laboratory of Applied Surface and Colloid Chemistry Ministry of Education, Shaanxi Provincial Basic Discipline (Surface and Interface Chemistry) Research Center, Shaanxi Key Laboratory for Advanced Energy Devices, Shaanxi Engineering Lab for Advanced Energy Technology, School of Materials Science and Engineering, Shaanxi Normal University, Xi’an 710119, P. R. China

^2^School of Science, Xi’an University of Posts & Telecommunications, Xi’an 710121, P. R. China

^3^State Key Laboratory of Radiation Medicine and Protection, School of Radiation Medicine and Protection and Collaborative Innovation Center of Radiation Medicine of Jiangsu Higher Education Institutions, Soochow University, Suzhou 215123, P. R. China

^4^State Key Laboratory of Catalysis, Dalian National Laboratory for Clean Energy, Dalian Institute of Chemical Physics, Chinese Academy of Sciences, Dalian 116023, P. R. China

^5^Center of Materials Science and Optoelectronics Engineering, University of Chinese Academy of Sciences, Beijing 100049, P. R. China

^#^Yujia Jiang and Depeng Chu contributed equally to this work.

*Corresponding authors. E-mail: [liuyc@snnu.edu.cn](mailto:liuyc@snnu.edu.cn) (Yucheng Liu); [yhhe@suda.edu.cn](mailto:yhhe@suda.edu.cn) (Yihui He); [szliu@dicp.ac.cn](mailto:szliu@dicp.ac.cn) (Shengzhong (Frank) Liu)

**S1 Characterizations and Methods**

**Theoretical calculation**

All quantum chemical calculations were conducted using Gaussian 16 A.03 software [S1]. The PBE0 functional was applied in combination with the Grimme D3(BJ) dispersion correction for all computations [S2-S4]. Geometry optimizations and frequency analyses were performed employing the def2-SVPbasis set for all atoms [S5, S6], while single-point energy calculations were carried out using the larger def2-TZVPP basis set [S5, S6]. To account for solvation effects in DMSO, the Solvation Model based on Solute Electron Density (SMD) implicit solvation model was incorporated during the single-point energy evaluations [S7]. The Gibbs free energy (G) and enthalpy (H) at 298.15 K were subsequently obtained using the Shermo software [S8].

Theoretical models were constructed according to the experimental feed ratio of CsCl:PbCl_2_: amine salt (MAI/MABr/MACl) = 2:3:2. Solvation was represented by three DMSO molecules per solute unit to approximate the primary solvent shell. The systems investigated together with their corresponding molecular ratios include: CsCl-PbCl_2_ (2:3), CsCl-PbCl_2_-DMSO (2:3:3), and CsCl-PbCl_2_-MAI/MABr/MACl-DMSO (2:3:3:3). The binding energy of each system was defined as the Gibbs free energy difference between the complex and its isolated components. For instance, the binding energy of the CsCl-PbCl_2_-MAI-DMSO system is calculated as:

$${\Delta G}_{(CsCl-{PbCl}_{2}-MAI-DMSO)}=G_{(CsCl-{PbCl}_{2}-MAI-DMSO)}-2G_{(CsCl)}-3G_{({PbCl}_{2})}-2G_{(MAI)}-3G_{(DMSO)}$$

**Material** **characterizations**

X-ray diffraction (XRD) patterns of the SCs were measured on a Rigaku MiniFlex 600 X-ray diffractometer with a Cu tube, the tube was operated at an acceleration voltage of 40 kV and a beam current of 20 mA. High-resolution XRD rocking curves were measured using an X’Pert pro MRD X-ray diffractometer (Cu-target) which was operated at 40 kV and 20 mA. X-ray photoelectron spectroscopy (XPS) measurements were carried out using an Escalab 250Xi spectrometer (Thermo Fisher Scientific). ^1^H nuclear magnetic resonance (NMR) measurements were employed to analyze the C-H bonding environments by using a Bruker Avance HD X500 spectrometer operating at 500 MHz. Absorbance spectra of the SCs were measured by using a Shimadzu UV-Vis-NIR spectrometer (Shimadzu, UV-3600). The steady-state and time-resolved photoluminescence characteristics of CsPbCl_3_ SC were investigated using a Pico-Quant FT-100. Thermogravimetric (TG) and differential scanning calorimetry (DSC) were performed using a TA SDT-Q600 V20.9 (Build 20) instrument. The SEM images were measured using a field emission SEM (Hitachi, SU-8020) and the compositions of the SCs were measured employing the energy-dispersive X-ray spectroscopy (EDX) of the SU-8020 (Shimadzu, EDX-7000).

**Relative dielectric constant (ε) measurements**

The capacitance-frequency curves of the SCs were measured using an impedance analyzer (Agilent 4980A Precision LCR Meter) over a wide frequency range from 0.03 MHz to 10 MHz to determine the capacitance of the SCs. The relative permittivity (ε) was calculated by the following equation:

$$\varepsilon=\frac{cd}{\varepsilon_{0}A}$$

where d and A are the thickness and the electrode area respectively, and ε_0_ is the vacuum permittivity.

**Space-charge-limited current (SCLC) measurements**

The dark current-voltage characteristics of the SC detetors were measured using a precision source meter (Keysight Technologies, B2902A). The experimental I-V datas were analyzed within the framework of the widely used space-charge-limited current (SCLC) model. By fitting the measured curves to the SCLC equation, the trap density of states (n_trap_) of the SCs was quantitatively extracted. Specifically, in the linear ohmic region of the dark current-voltage curve, the average trap density was calculated according to the following formula:

$$n_{trap}=\frac{2\varepsilon\varepsilon_{0}V_{TEL}}{qL^{2}}$$

where ε is the dielectric constant and ε_0_ the vacuum permittivity. q is the elementary charge, L the thickness of the SC, and V_TFL_ the trap-filled limit voltage. The V_TFL_ can be read directly from the dark current-voltage curve, therefore the trap density can be calculated.

**Mobility of hole carriers measurements**

Mobility of hole carriers were assessed using ~1 μCi ^241^Am (5.5 MeV) α particle and ~10 μCi ^57^Co (122 keV) γ-ray sources. Bismuth (Bi) and gold (Au) electrodes were prepared via thermal evaporation with the thickness of 70 nm. Both electrodes were connected to Cu wires using Ag paint and then to outer readout circuits. The as-fabricated devices placed in a shielding box were connected to an eV-550 preamplifier. The negative bias voltage was applied on the Au electrode via the eV-550 preamplifier using ORTEC 710 Bias Supply and the alpha particles and γ-rays were irradiated from Bi electrode. The induced signals from the device were further amplified using an ORTEC model 572A amplifier with a shaping time constant of 10 μs. The shaped signals were then digitized by a dual 16 K input multichannel analyzer (ORTEC-927), finally generating a response spectrum displayed on MAESTRO-32 software.

**Photoconductivity measurements**

For photoconductivity measurements, X-rays were used for illumination light. The I-V curves of the SC detectors under X-ray irradiation were measured and the X-ray response current was fitted using the modified Hecht equation to derive the *μτ* product with the following formula:

$$I=\frac{I_{o}\mu\tau V}{L^{2}}\frac{1-exp(-\frac{L^{2}}{\mu\tau V})}{1+\frac{L}{V}\frac{s}{\mu}}$$

where *I*_0_ is the saturated photocurrent, *L* is the thickness of the SC detector, *V* is the applied bias and *τ* is the carrier lifetime.

**Detection limit evaluation by the signal-to-noise ratio (SNR) of the SC X-ray detectors** According to measuring the response current of the SC detector under on-off X-rays, the average dark current and the average X-ray response current are obtained. Then we confirm the dose rate corresponding to an output with a signal-to-noise ratio (SNR) of 3 as the limit of detection at a fixed bias and the SNR is calculated as follows:

$$SNR= \frac{I_{signal}}{I_{noise}}$$

where the signal current (*I*_signal_) is calculated by subtracting the average dark current ($\bar{I}_{dark}$) from the average X-ray response current ($\bar{I}_{photo}$) detected by the detector. The noise current (*I*_noise_) was obtained by calculating the standard deviation of the x-ray response current:

$$I_{noise}= \sqrt{\frac{1}{N} \sum_{i}^{N} {(I_{i}-\bar{I}_{photo})}^{2}}$$

Finally, we measured the X-ray response current over time at different dose rates to determine the detection limit.

**X-ray detector performance measurements**

All X-ray response measurements of the CsPbCl_3_ SC detectors were conducted at room temperature under ambient atmospheric conditions. The detector was enclosed within a custom-fabricated lead-shielding chamber to suppress electromagnetic interference, eliminate stray optical illumination, and reduce radiation exposure to operators. X-ray irradiation was provided by a tungsten-anode source (Spellman, XRB150PN600) operated at an accelerating voltage of 70 kV. The tube current was systematically varied in the range of 1-3.5 mA to control the incident dose rate. Additional dose-rate modulation was achieved by introducing five aluminum attenuators with a thickness of 3 mm between the source and the detector. The absolute dose rate was calibrated using a high-precision silicon diode dosimeter (RaySafe-X2, Fluke). The photocurrent response time of the SC detector was measured by a precision source meter (FS-Pro). To evaluate the temporal response characteristics, pulsed X-rays of variable frequency were generated using a lead chopper.

**X-ray imaging**

X-ray imaging experiments were conducted using a dedicated system composed of a lead-shielded enclosure, a tungsten-anode X-ray source, a motorized X-Y translation stage (Thorlabs, MLS203-1) and a precision source meter (Keysight B2911B). The specimen was mounted on the translation stage which provided controlled bidirectional displacement under continuous X-ray irradiation. The photocurrent of the SC detector was simultaneously acquired by the precision source meter in correspondence with the stage coordinates, enabling precise two-dimensional scanning. The resulting spatially correlated data were used to reconstruct high-resolution X-ray transmission images of the specimen.

**S2 Supplementary Figures and Tables**

**
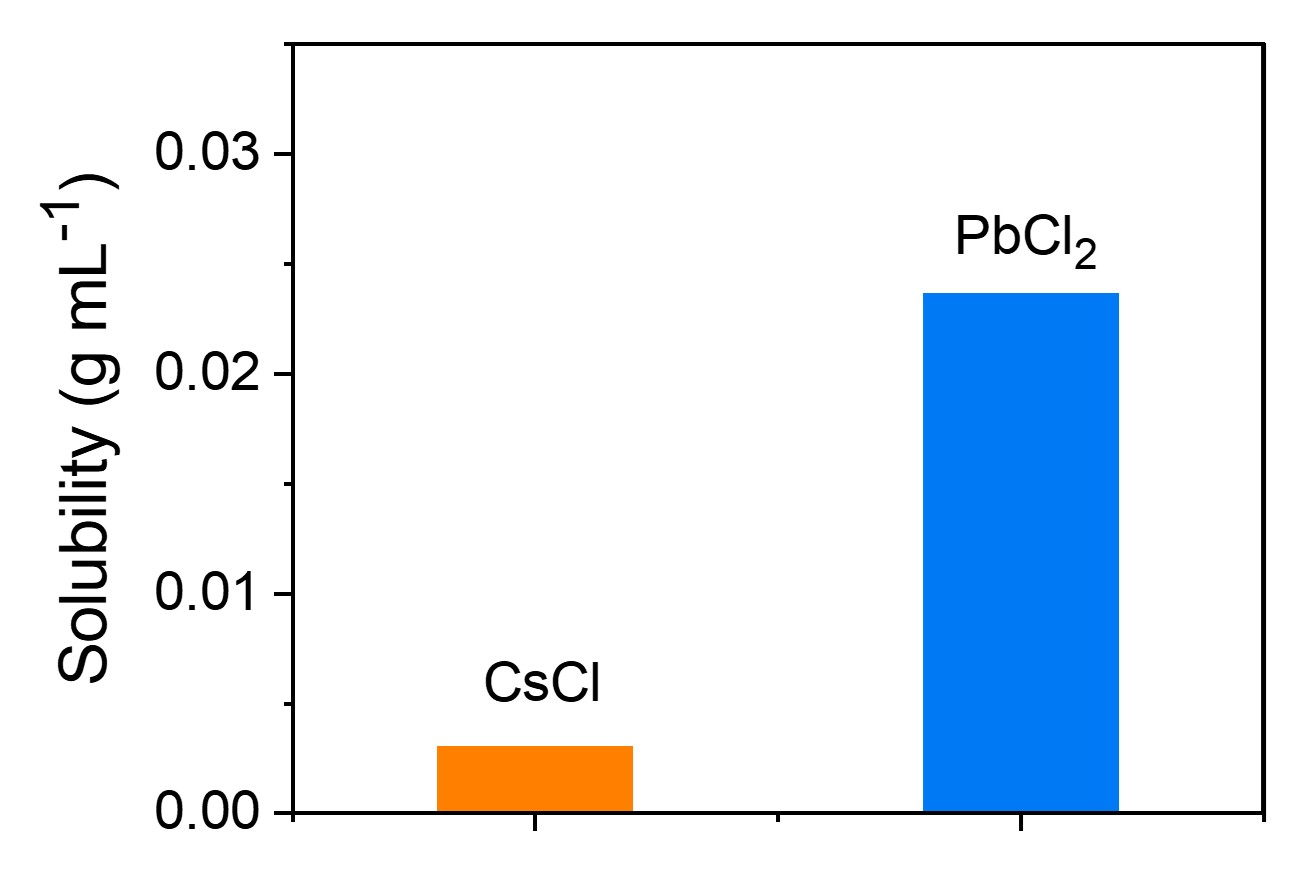
**

**Fig. S1** Solubility of CsCl and PbCl_2_ in DMSO solvent at room temperature.

**

**

**Fig. S2** The structural formulas of monomers and coordinated systems used for energy calculation. **a-f** Monomer structures of CsCl, PbCl_2_, DMSO, MAI, MABr and MACl. **g-j** The coordinated structures of CsCl-PbCl_2_-DMSO, CsCl-PbCl_2_-DMSO-MAI, CsCl-PbCl_2_-DMSO-MABr and CsCl-PbCl_2_-DMSO-MACl systems.


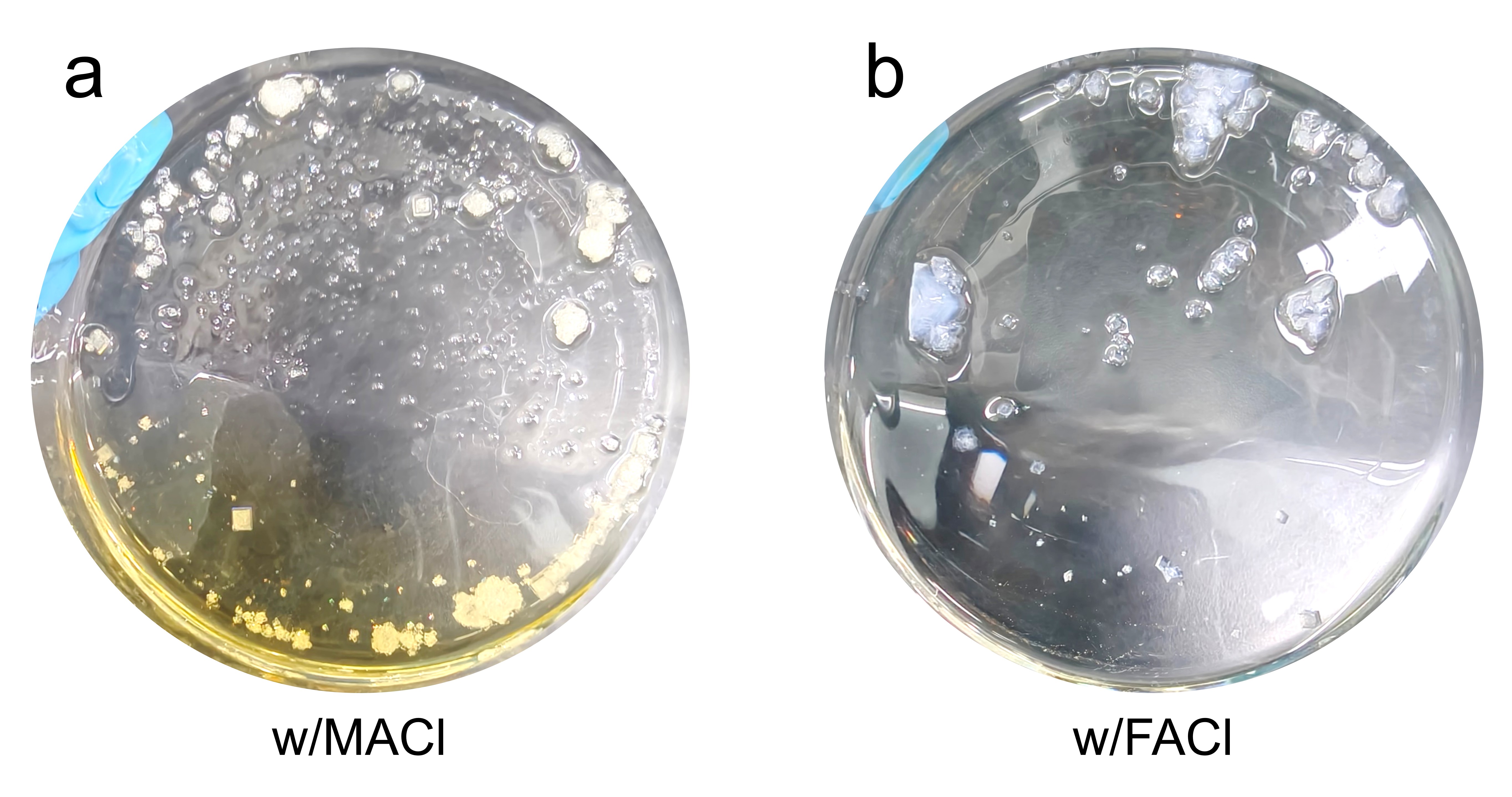


**Fig. S3** Photographs of single crystals grown in the solution with different additives. **a** With MACl. **b** With FACl.


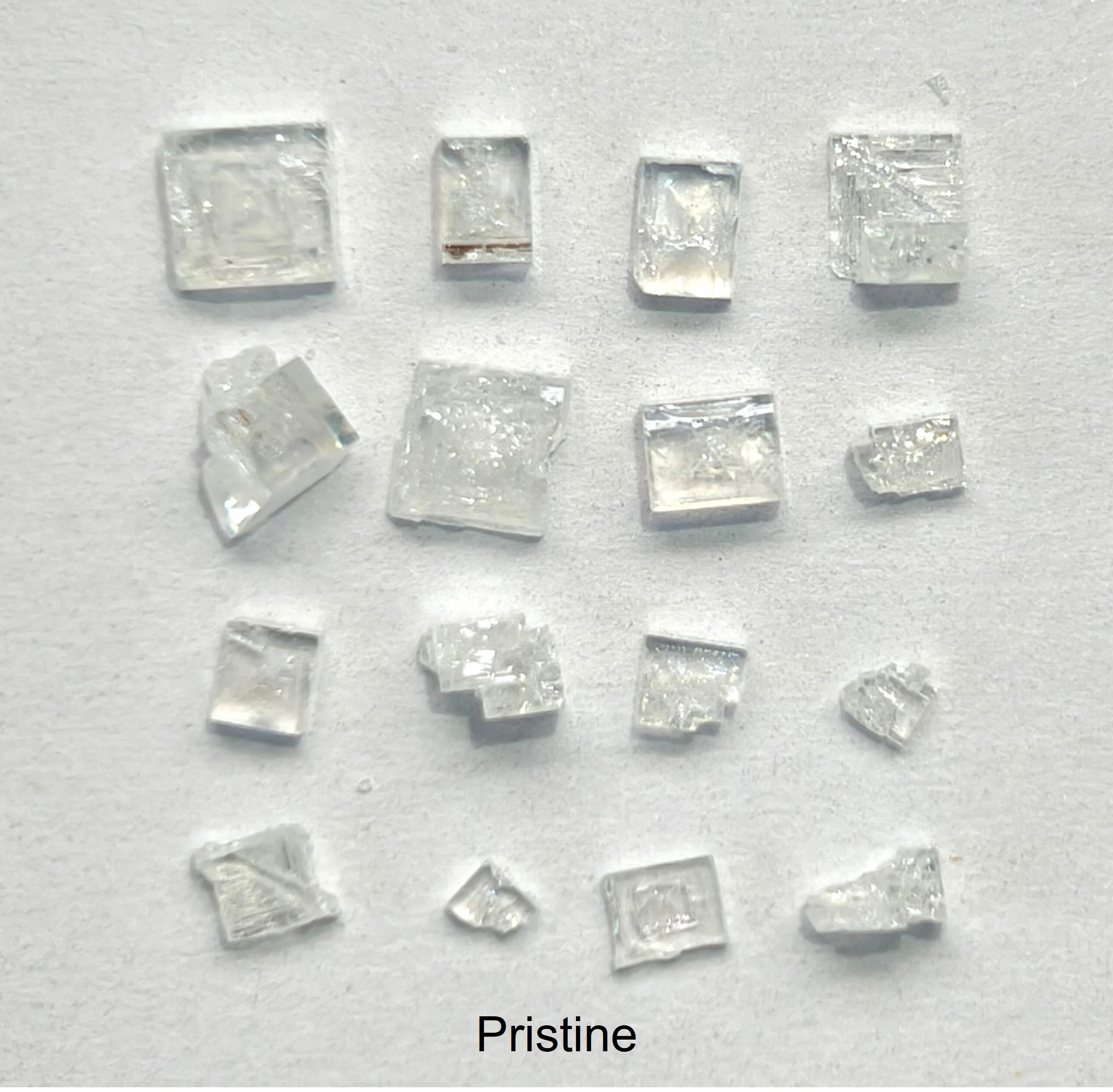


**Fig. S4** Photographs of CsPbCl_3_ SCs grown in the pristine solution (without additives). Many obvious cracks can be recognized inside in the SCs.


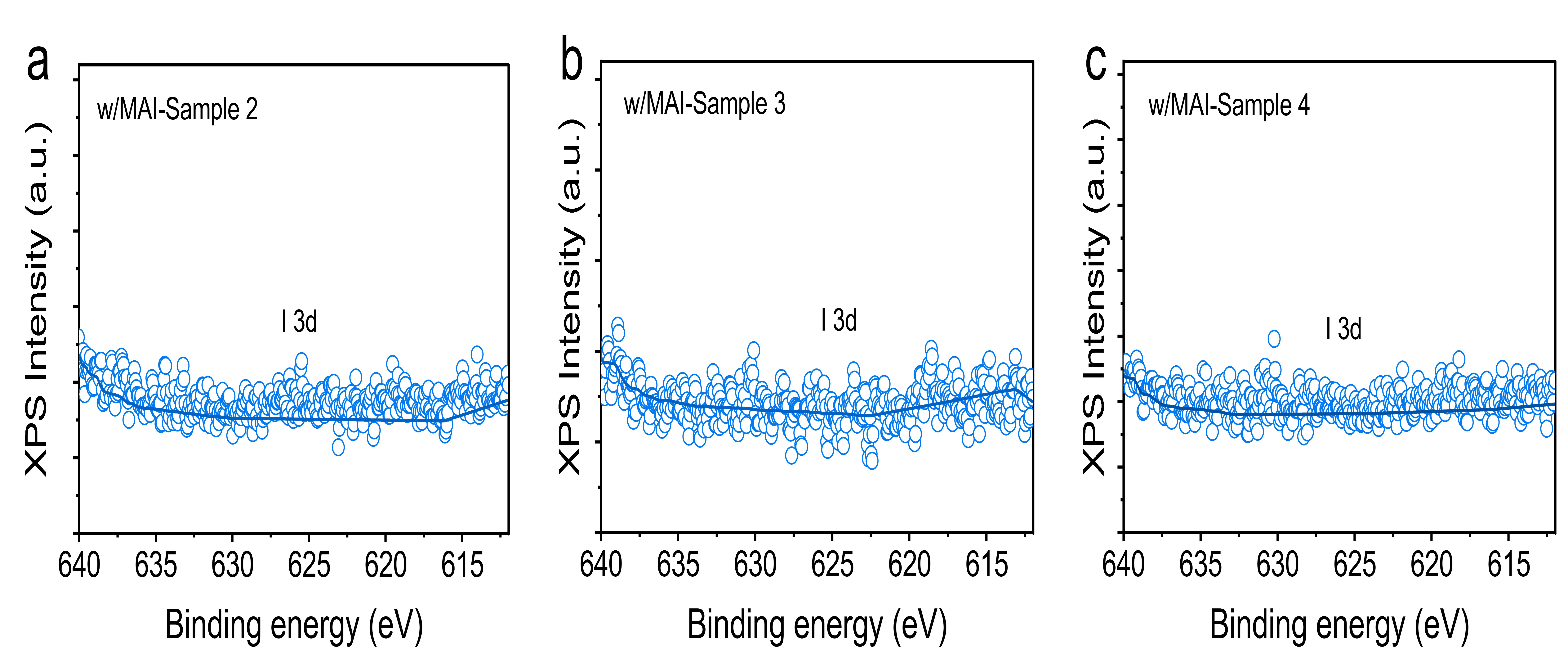


**Fig. S5** High-resolution XPS spectra of I 3d for the CsPbCl_3_ SCs grown from the solution with MAI.





**Fig. S6** Elemental composition and distribution in the SCs grown from the solution with MAI. **a-c** XPS spectra of Cs 3d, Pb 4f, and Cl 2p of the SC. **d** SEM images of the SC. **e-g** SEM-EDS mapping of Cs, Pb, Cl for the SC. **h** Energy-dispersive X-ray spectra of the SC.





**Fig. S7** Elemental composition and distribution in the SCs grown from the solution with FAI. **a-e** XPS spectra of Cs 3d, Pb 4f, Cl 2p and I 3d of the SC. **f** SEM images of the SC. **g-i** SEM-EDS mapping of Cs, Pb, Cl for the SC. **j** Energy-dispersive X-ray spectra of the SC. **k** Comparison of calculated Cs:Pb:Cl ratio in the SC with experimental results measured by XPS and EDS, respectively.





**Fig. S8** Elemental composition and distribution in the SCs grown from the solution with MABr. **a-e** XPS spectra of Cs 3d, Pb 4f, Cl 2p and Br 3d of the SC. **f** SEM images of the SC. **g-j** SEM-EDS mapping of Cs, Pb, Cl and Br for the SC. **k** Energy-dispersive X-ray spectra of the SC. **l** Comparison of calculated Cs:Pb:Cl ratio in the SC with experimental results measured by XPS and EDS, respectively.





**Fig. S9 Elemental composition and distribution in the SCs grown from the solution with FABr.** **a-e** XPS spectra of Cs 3d, Pb 4f, Cl 2p and Br 3d of the SC. **f** SEM images of the SC. **g-j** SEM-EDS mapping of Cs, Pb, Cl and Br for the SC. **k** Energy-dispersive X-ray spectra of the SC. **l** Comparison of calculated Cs:Pb:Cl ratio in the SC with experimental results measured by XPS and EDS, respectively.


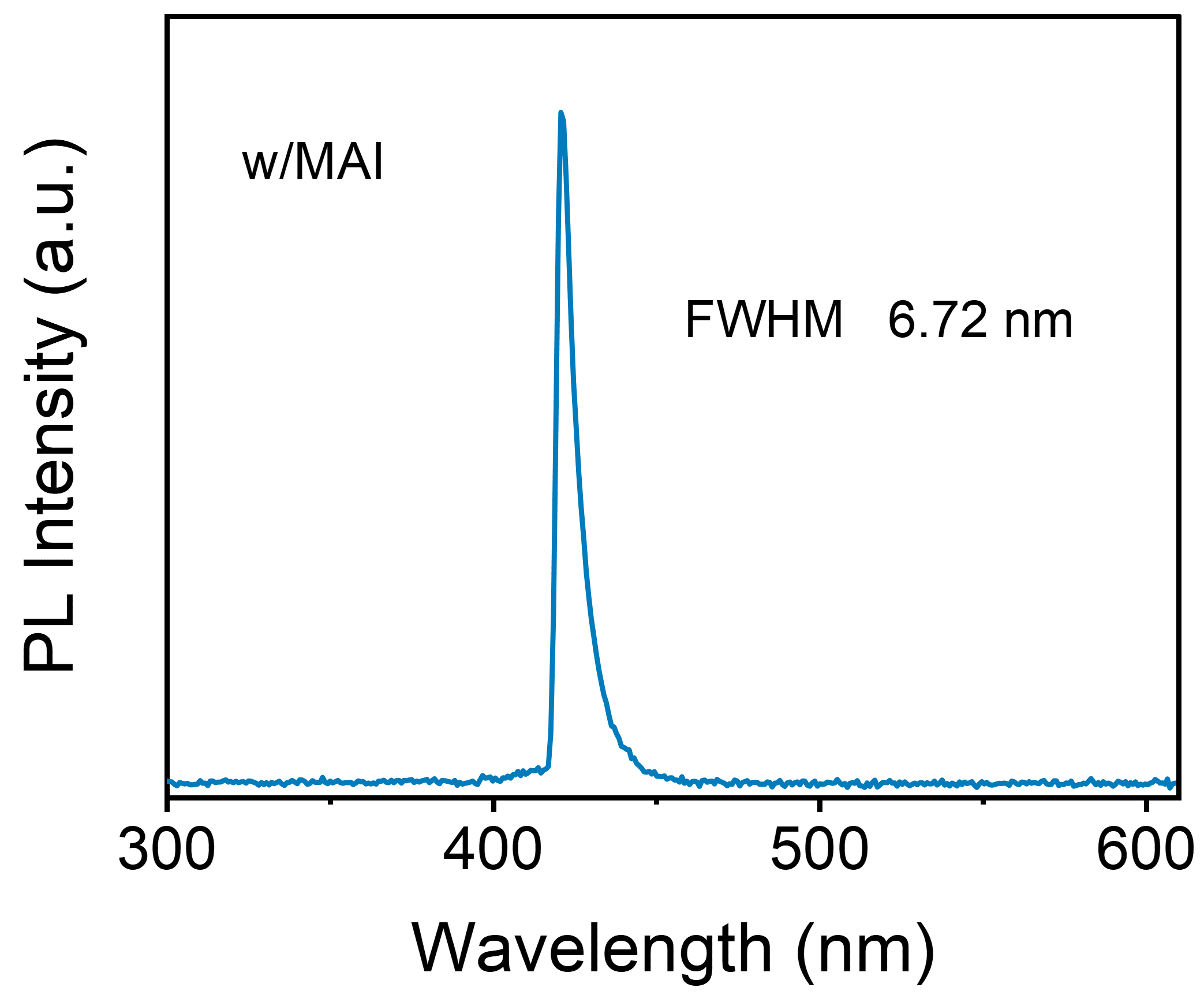


**Fig. S10** Photoluminescence spectrum of the CsPbCl_3_ SC.


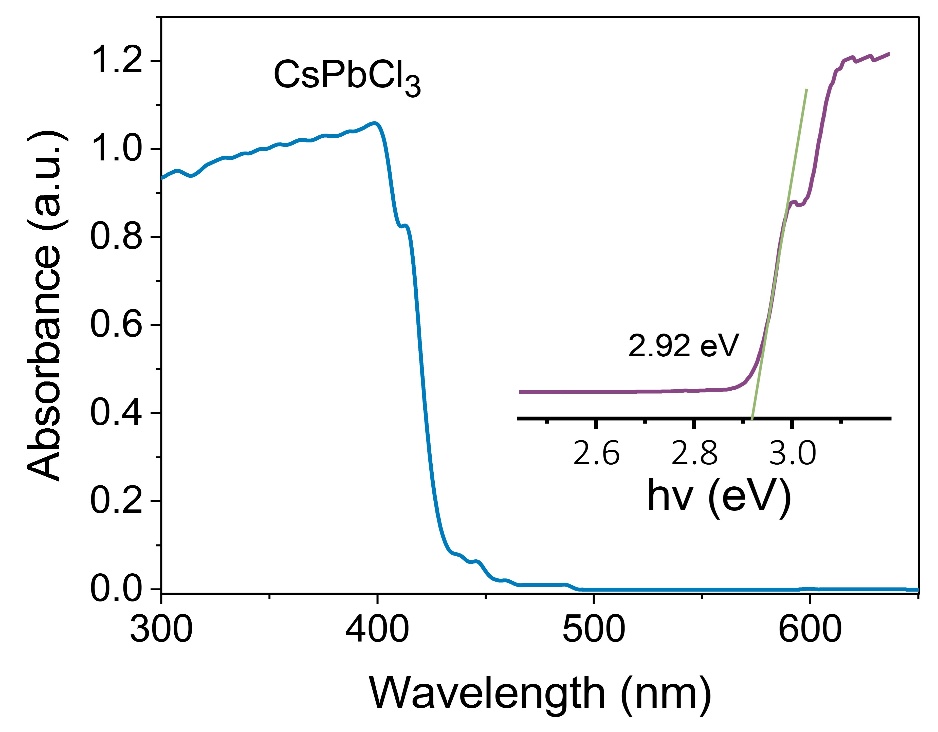


**Fig. S11** Absorption spectrum and corresponding band gap calculation of the CsPbCl_3_ SC grown from the solution with MAI.


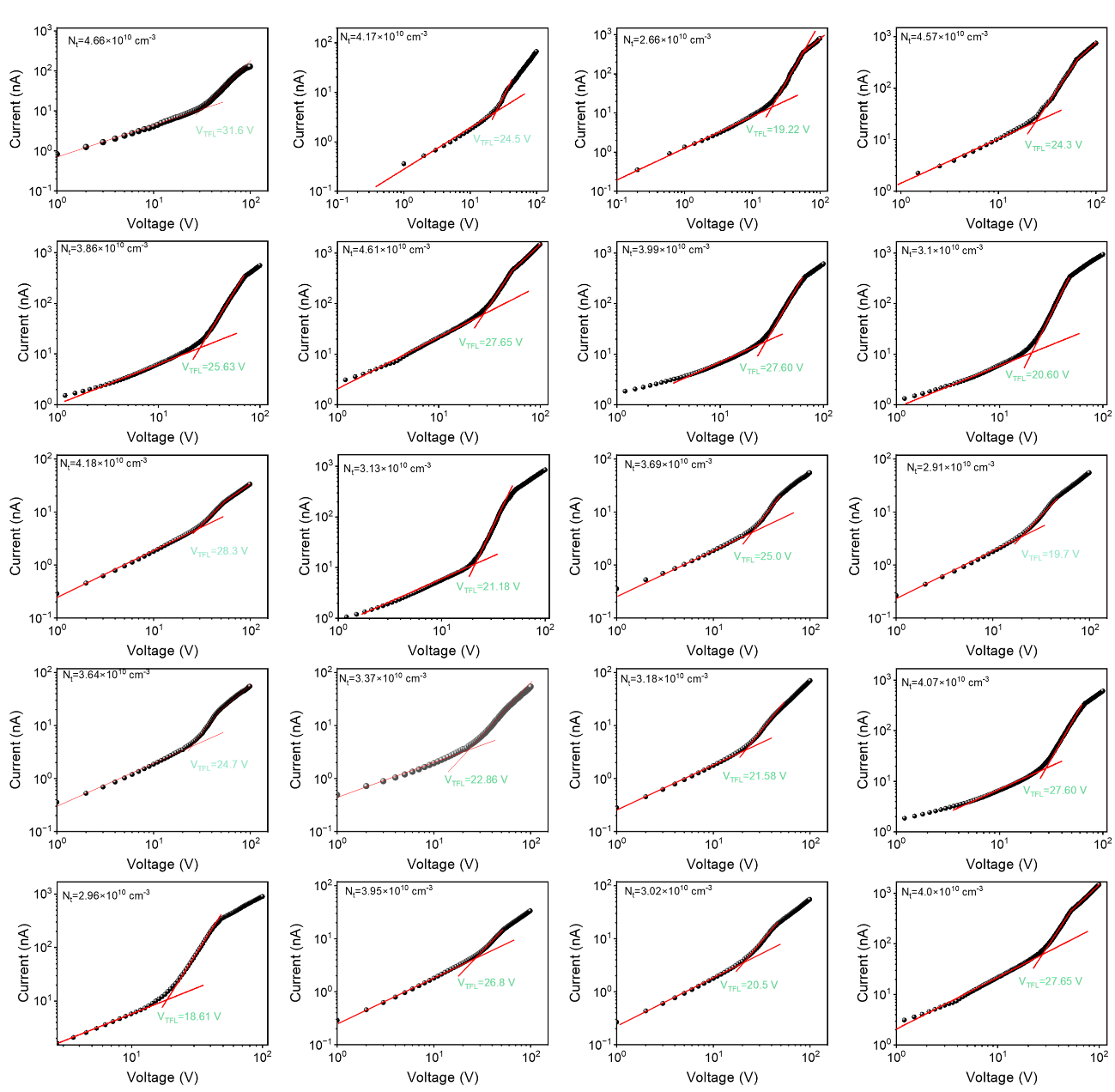


**Fig. S12** Dark current-voltage (I-V) curves of the CsPbCl_3_ SCs.


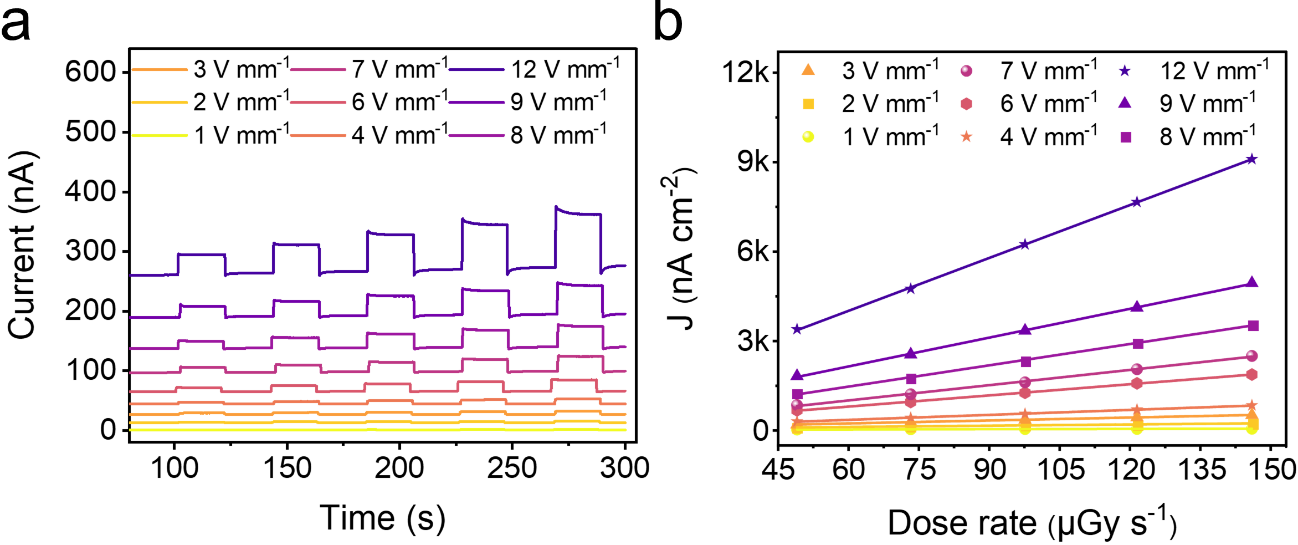


**Fig. S13** X-ray response current of the CsPbCl_3_ SC detector. **a** Response current of the SC detector under on−off X-rays with different dose rates. **b** Dose rate dependence response of the SC detector under X-ray irradiation at different electric fields.


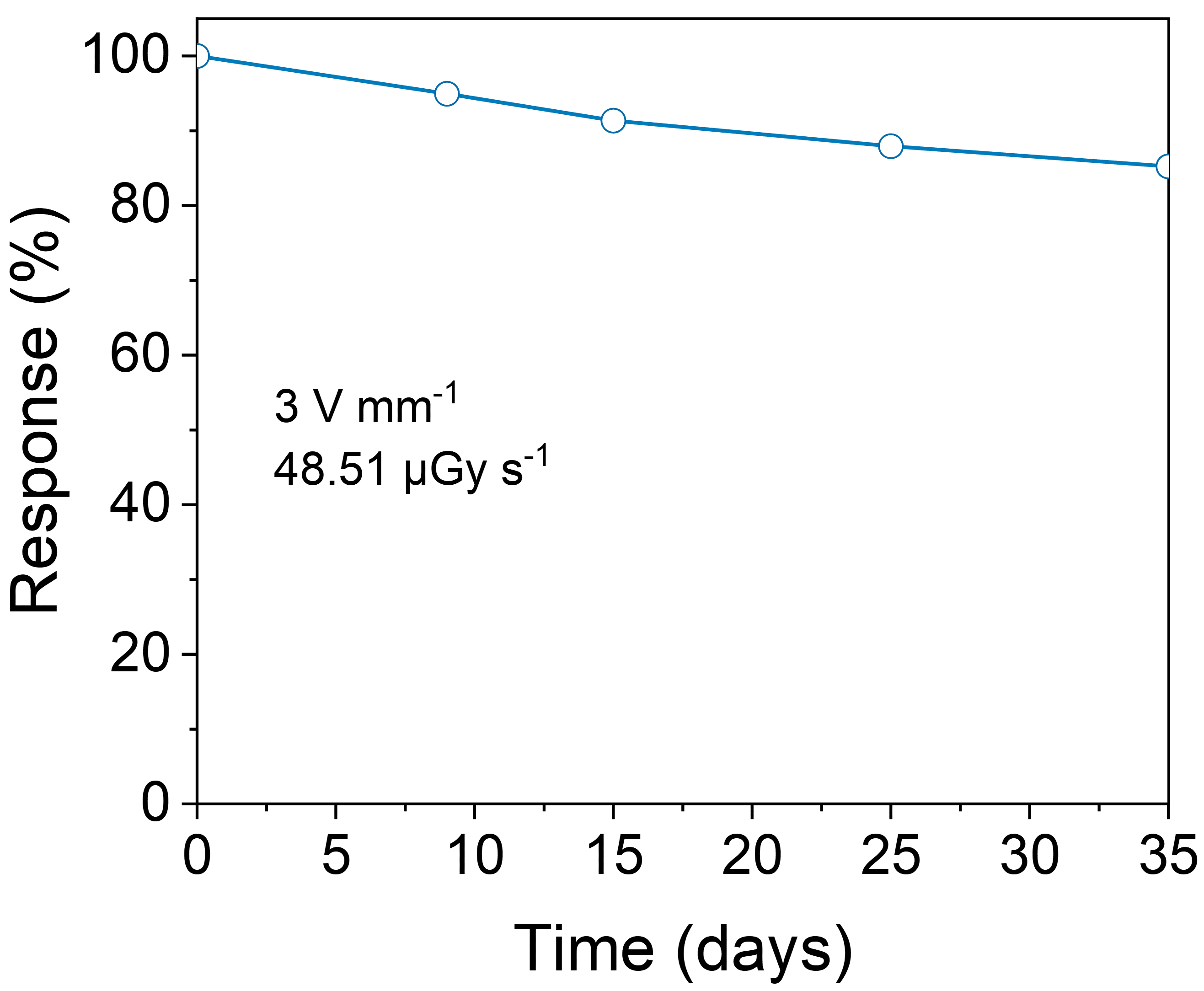


**Fig. S14** Normalized X-ray response of the CsPbCl_3_ SC detector after storage for 35 days in air environment.

**Table S1** Theoretical calculations of optimized total electronic and Gibbs free energies of individual molecular species

| **Molecular species** | **Gibbs free energy (G)**  **(kcal mol^-1^)** |
| --- | --- |
| DMSO | -346973.34 |
| CsCl | -301436.3 |
| PbCl_2_ | -698513.59 |
| MAI | -246908.9 |
| MABr | -1675200.83 |
| MACl | -348766.57 |

**Table S2** Theoretical optimized total electronic and Gibbs free energies of multi-component molecular complexes

| **Molecular complex** | **Optimized total electronic energy (E)**  **(kcal·mol^-1^)** | **Gibbs free energy (G)**  **(kcal mol^-1^)** |
| --- | --- | --- |
| CsCl-PbCl_2_ | -2698334.28 | -2698413.37 |
| CsCl-PbCl_2_-DMSO | -3742470.53 | -3739399.36 |
| CsCl-PbCl_2_-MACl-DMSO | -4432179.17 | -4436938.49 |
| CsCl-PbCl_2_-MABr-DMSO | -7099577.83 | -7089809.64 |
| CsCl-PbCl_2_-MAI-DMSO | -4236590.38 | -4233228.83 |

**Table S3** The Gibbs free energy change of the calculated multi-component molecular complexes

| **Molecular complex** | **ΔG (kcal mol^-1^)** |
| --- | --- |
| CsCl-PbCl_2_-DMSO | -65.97 |
| CsCl-PbCl_2_-MACl-DMSO | -71.96 |
| CsCl-PbCl_2_-MABr-DMSO | -74.59 |
| CsCl-PbCl_2_-MAI-DMSO | -77.64 |

**Table S4** Comparison of the key parameters of the SC X-ray detector in this study and other detectors

| **Materials** | **Sensitivity**  **(μC Gy^-1^ cm^2^)** | **Electric field or voltage** | ***μτ* product**  **(cm^2^ V^-1^ s^-1^)** | **Detection limit**  **(nGy s^-1^)** | **Electric field or voltage** | **Ref.** |
| --- | --- | --- | --- | --- | --- | --- |
| CsPbCl_3_ SC | 76,624 | 20 V mm^-1^ | 6.3 × 10^-3^ | 47.9 | 1 V mm^-1^ | This work |
| Cs_3_Bi_2_I_9_ SC | 1,652.3 | 50 V mm^-1^ | 7.97 × 10^-4^ | 130 | 50 V mm^-1^ | [S9] |
| Cs_3_Bi_2_I_9_ SC | 59 464.4 | 150 V | 5.53 × 10^-4^ | 1.27 | 5 V | [S10] |
| Cs_2_AgBiBr_6_ SC | 105.0 | 50 V | 6.3 × 10^-3^ | 59.7 | 5 V | [S11] |
| Cs_4_PbI_6_ SC | 451.5 | 30 V | 9.7 × 10^-4^ | - | - | [S12] |
| CsPbBr_3_ Film | 1,450 | 300 V | - | 500 | 100 V | [S13] |
| CsPbBr_3_ SC | 4086 | 5 V mm^-1^ | - | - | - | [S14] |
| CsPbBr_3_ SC | 6021.99 | 50 V | 5.14 × 10^-3^ | 1890 | 5 V | [S15] |
| CsPbBr_3_ SC | 10,833 | 435 V mm^-1^ | 9.4 × 10^-4^ | 32 | 435 V mm^-1^ | [S16] |
| CsPbBr_3_ film | 55,684 | 5 V mm^-1^ | 1.32 × 10^-2^ | 215 | ~4.2 V mm^-1^ | [S17] |
| CsPbBr_3_ SC | 46180 | 500 V mm^-1^ | 8.11 × 10^-4^ | 10.81 | - | [S18] |
| CsPbBr_3_ SC | 55722 | 300 V mm^-1^ | 2.4 × 10^-4^ | - | - | [S19] |
| CsPbCl_2.995_I_0.005_ SC | 2.19 × 10^5^ | 100 V mm^-1^ | 1.02 × 10^-3^ | - | - | [S20] |
| α-Se | ~20 | 10000 V mm^-1^ | - | - | - | [S21] |
| CZT | 20 | 250 V mm^-1^ | - | 145 | - | [S22] |

**Supplementary References**

1. M. Frisch, G. Trucks, H. Schlegel, G. Scuseria, M. Robb et al., Gaussian 09, Revision C.01. Gaussian, Inc., Wallingford, CT (2016).
2. C. Adamo, V. Barone, Toward reliable density functional methods without adjustable parameters: The PBE0 model. J. Chem. Phys. **110**(13), 6158–6170 (1999). <https://doi.org/10.1063/1.478522>
3. S. Grimme, Density functional theory with London dispersion corrections. Wires Comput. Mol. Sci. **1**(2), 211–228 (2011). <https://doi.org/10.1002/wcms.30>
4. S. Grimme, S. Ehrlich, L. Goerigk, Effect of the damping function in dispersion corrected density functional theory. J. Comput. Chem. **32**(7), 1456–1465 (2011). <https://doi.org/10.1002/jcc.21759>
5. F. Weigend, R. Ahlrichs, Balanced basis sets of split valence, triple *Zeta* valence and quadruple *Zeta* valence quality for H to Rn: Design and assessment of accuracy. Phys. Chem. Chem. Phys. **7**(18), 3297–3305 (2005). <https://doi.org/10.1039/b508541a>
6. F. Weigend, Accurate coulomb-fitting basis sets for H to Rn. Phys. Chem. Chem. Phys. **8**(9), 1057–1065 (2006). <https://doi.org/10.1039/b515623h>
7. A.V. Marenich, C.J. Cramer, D.G. Truhlar, Universal solvation model based on solute electron density and on a continuum model of the solvent defined by the bulk dielectric constant and atomic surface tensions. J. Phys. Chem. B **113**(18), 6378–6396 (2009). <https://doi.org/10.1021/jp810292n>
8. T. Lu and Q. Chen, Interaction region indicator: A simple real space function for identifying interaction regions from molecular wavefunctions and electron density. Comput. Theor. Chem. 1200, 113249 (2021). <https://doi.org/10.1016/j.comptc.2021.113249>
9. Y. Zhang, Y. Liu, Z. Xu, H. Ye, Z. Yang et al., Nucleation-controlled growth of superior lead-free perovskite Cs_3_Bi_2_I_9_ single-crystals for high-performance X-ray detection. Nat. Commun. **11**, 2304 (2020). <https://doi.org/10.1038/s41467-020-16034-w>
10. M. Yang, A. Li, X. Hao, L. Wu, W. Tian et al., Highly sensitive X-ray detector made of large lead-free perovskite Cs_3_Bi_2_I_9_ single crystals with anisotropic response. Adv. Opt. Mater. **11**(15), 2203066 (2023). <https://doi.org/10.1002/adom.202203066>
11. W. Pan, H. Wu, J. Luo, Z. Deng, C. Ge et al., Cs_2_AgBiBr_6_ single-crystal X-ray detectors with a low detection limit. Nat. Photonics **11**(11), 726–732 (2017). <https://doi.org/10.1038/s41566-017-0012-4>
12. Q. Xu, C. Li, J. Nie, Y. Guo, X. Wang et al., Highly sensitive and stable X-ray detector based on a 0D structural Cs_4_PbI_6_ single crystal. J. Phys. Chem. Lett. **12**(1), 287–293 (2021). <https://doi.org/10.1021/acs.jpclett.0c03411>
13. G.J. Matt, I. Levchuk, J. Knüttel, J. Dallmann, A. Osvet et al., Sensitive direct converting X-ray detectors utilizing crystalline CsPbBr_3_ perovskite films fabricated *via* scalable melt processing. Adv. Mater. Interfaces **7**(4), 1901575 (2020). <https://doi.org/10.1002/admi.201901575>
14. J. Peng, C.Q. Xia, Y. Xu, R. Li, L. Cui et al., Crystallization of CsPbBr_3_ single crystals in water for X-ray detection. Nat. Commun. **12**(1), 1531 (2021). <https://doi.org/10.1038/s41467-021-21805-0>
15. J. Di, H. Li, J. Su, H. Yuan, Z. Lin et al., Reveal the humidity effect on the phase pure CsPbBr_3_ single crystals formation at room temperature and its application for ultrahigh sensitive X-Ra*_y_* detector. Adv. Sci. **9**(2), 2103482 (2022). <https://doi.org/10.1002/advs.202103482>
16. L. Pan, Z. Liu, C. Welton, V.V. Klepov, J.A. Peters et al., Ultrahigh-flux X-ray detection by a solution-grown perovskite CsPbBr_3_ single-crystal semiconductor detector. Adv. Mater. **35**(25), 2211840 (2023). <https://doi.org/10.1002/adma.202211840>
17. W. Pan, B. Yang, G. Niu, K.-H. Xue, X. Du et al., Hot-pressed CsPbBr_3_ quasi-monocrystalline film for sensitive direct X-ray detection. Adv. Mater. **31**(44), 1904405 (2019). <https://doi.org/10.1002/adma.201904405>
18. Y. Hua, G. Zhang, X. Sun, P. Zhang, Y. Hao et al., Suppressed ion migration for high-performance X-ray detectors based on atmosphere-controlled EFG-grown perovskite CsPbBr_3_ single crystals. Nat. Photonics **18**(8), 870–877 (2024). <https://doi.org/10.1038/s41566-024-01480-5>
19. X. Sun, G. Zhang, W. Ma, Y. Hua, H. Liu et al., Growth of two-inch perovskite CsPbBr_3_ single-crystal with high irradiation resistance for X-ray detection. Adv. Mater. **38**(1), e12788 (2026). <https://doi.org/10.1002/adma.202512788>
20. J. Tian, R. Shi, D. Chu, N. Liu, J. Pi et al., Portable dosimeter with ultralow detection limit enabled by large size solution grown inorganic perovskite single crystal. Adv. Mater. **38**(18), e22163 (2026). <https://doi.org/10.1002/adma.202522163>
21. S. Kasap, J.B. Frey, G. Belev, O. Tousignant, H. Mani et al., Amorphous selenium and its alloys from early xeroradiography to high resolution X-ray image detectors and ultrasensitive imaging tubes. Phys. Status Solidi B: . **246**(8), 1794–1805 (2009). <https://doi.org/10.1002/pssb.200982007>
22. S. Tokuda, H. Kishihara, S. Adachi, T. Sato, Preparation and characterization of polycrystalline CdZnTe films for large-area, high-sensitivity X-ray detectors. J. Mater. Sci. Mater. Electron. **15**(1), 1–8 (2004). <https://doi.org/10.1023/A:1026297416093>
